# Efficient Matrix Cleanup of Soft-Gel-Type Dietary Supplements for Rapid Screening of 92 Illegal Adulterants Using EMR-Lipid dSPE and UHPLC-Q/TOF-MS

**DOI:** 10.3390/ph14060570

**Published:** 2021-06-15

**Authors:** Beom Hee Kim, Wonwoong Lee, You Lee Kim, Ji Hyun Lee, Jongki Hong

**Affiliations:** 1 College of Pharmacy, Kyung Hee University, Seoul 02447, Korea; nypdmain@naver.com (B.H.K.); uiriuiri@naver.com (Y.L.K.); 2 College of Pharmacy, Woosuk University, Wanju 55338, Korea; wwlee@woosuk.ac.kr; 3 College of Pharmacy, Chungbuk National University, Osong 28160, Korea; ljhyune@korea.kr

**Keywords:** illegal adulterants, soft-gel, EMR-Lipid dSPE, dietary supplements, extracted common ion chromatogram, neutral loss scan, UHPLC-Q/TOF-MS

## Abstract

An efficient matrix cleanup method was developed for the rapid screening of 92 illegal adulterants (25 erectile dysfunction drugs, 15 steroids, seven anabolic steroids, 12 antihistamines, 12 nonsteroidal anti-inflammatory drugs (NSAIDs), four diuretics, and 17 weight-loss drugs) in soft-gel-type supplements by ultra-high performance liquid chromatography-quadrupole/time of flight-mass spectrometry (UHPLC-Q/TOF-MS). As representative green chemistry methods, three sample preparation methods (dispersive liquid-liquid microextraction (DLLME), “quick, easy, cheap, effective, rugged, and safe” dispersive solid-phase extraction (QuEChERS-dSPE), and enhanced matrix removal-lipid (EMR-Lipid) dSPE) were evaluated for matrix removal efficiency, recovery rate, and matrix effect. In this study, EMR-Lipid dSPE was shown to effectively remove complicated matrix contents in soft-gels, compared to DLLME and QuEChERS-dSPE. For the rapid screening of a wide range of adulterants, extracted common ion chromatogram (ECIC) and neutral loss scan (NLS) based on specific common MS/MS fragments were applied to randomly collected soft-gel-type dietary supplement samples using UHPLC-Q/TOF-MS. Both ECICs and NLSs enabled rapid and simple screening of multi-class adulterants and could be an alternative to the multiple reaction monitoring (MRM) method. The developed method was validated in terms of limit of detection (LOD), precision, accuracy, recovery, and matrix effects. The range of LODs was 0.1–16 ng/g. The overall precision values were within 0.09–14.65%. The accuracy ranged from 81.6% to 116.6%. The recoveries and matrix effects of 92 illegal adulterants ranged within 16.9–119.4% and 69.8–114.8%, respectively. The established method was successfully applied to screen and identify 92 illegal adulterants in soft-gels. This method can be a promising tool for the high-throughput screening of various adulterants in dietary supplements and could be used as a more environmentally friendly routine analytical method for screening dietary supplements illegally adulterated with multi-class drug substances.

## 1. Introduction

With the increased public interest in health over the past decade, the global market for dietary supplements has been grown exponentially [1]. However, to increase the efficacy of dietary supplements, they have been frequently adulterated with several types of chemicals, such as pharmaceuticals, unapproved drugs, prohibited ingredients, and their analogues [2,3,4,5,6]. In particular, illegally adulterated supplements without any label can lead to various side effects and they further advance a serious public health problem.

To satisfy the needs of manufacturers, distributors, and consumers, dietary supplements have been produced and distributed in various formulations, such as tablet, hard capsule, pill, powder, and soft-gel forms. For active ingredients with low aqueous solubility, soft-gel-type supplements have been widely used to increase bioavailability. As a representative oral dosage form, a soft-gel consists of gelatin shell and various fillings including oil solutions. Since oil suspensions in soft-gel fillings could frequently disturb the routine screening tests due to their highly complex matrices [7,8,9,10], soft-gel-type supplements have been adulterated with illegal adulterants. In particular, phosphodiesterase type-5 (PDE-5) inhibitors, steroids, weight-loss drugs, and their analogues have often been detected in soft-gel-type supplements [11,12,13].

To supervise and prevent illegal adulterations in dietary supplements, numerous screening methods have been developed to effectively detect a wide range of illegal adulterants in various supplement formulations. Liquid chromatography-tandem mass spectrometry (LC-MS/MS) and quadrupole/time of flight (Q/TOF) MS methods have most often been used for screening of a wide range of unauthorized drugs in various supplements due to their high sensitivity, selectivity, and convenience [14,15,16,17]. LC-MS methods with aid from multiple reaction monitoring (MRM), extracted ion chromatogram (EIC), extracted common ion chromatogram (ECIC), and neutral loss scan (NLS) modes have all facilitated multi-class drug screening for various types of supplement. Nevertheless, analyses of soft-gel supplements with viscous fillings are prone to inaccurate results owing to high matrix complexity [18].

The oil fillings of soft-gel-type supplements mainly include triglycerides, diglycerides, monoglycerides, free fatty acids, and fatty acid esters of hydroxyl compounds (such as ethyl alcohol, propylene glycol, glycerin, sorbitol, sucrose, and polyethylene glycol) [7]. The complicated matrix of lipid type soft-gels could lead to severe interference on extraction and detection of target analytes, and further cause signal suppression or enhancement. Similarly, without appropriate sample pretreatments, these matrix interferences could seriously degrade analytical performance on both qualitative and quantitative results. Therefore, a sample pretreatment method that removes matrix interference materials without any significant loss of target analyte is an essential prerequisite to achieve reliable screening results for illegal adulterants in soft-gel-type dietary supplements.

Among various sample preparation methods, solvent extraction (SE) [16], “quick, easy, cheap, effective, rugged, and safe” dispersive solid-phase extraction (QuEChERS-dSPE) [19,20,21,22], and dispersive liquid-liquid microextraction (DLLME) [19,20,23] have been applied for the analysis of multi-class analytes such as pharmaceuticals and pesticides. Taking into consideration greener chemistry methodology, simple and convenient QuEChERS-dSPE and DLLME have been frequently employed to decrease use of organic solvents compared to SE method. The QuEChERS-dSPE and DLLME methods provide several advantages such as small-scale sample preparation, use of small amounts of organic solvents, and comprehensive extraction of a wide range of target analytes. Furthermore, as a modified QuEChERS-dSPE method, enhanced matrix removal (EMR)-lipid dSPE has been employed to efficiently remove lipid components in samples [24,25,26]. Although the composition of the EMR-Lipid kit has not been disclosed, a fundamental mechanism of lipid cleanup might be related to size exclusion and hydrophobic interactions [27].

In this study, a UHPLC-Q/TOF-MS method combined with EMR-Lipid dSPE was developed to simultaneously analyze 92 illegal adulterants in soft-gel-type dietary supplements. The DLLME, QuEChERS-dSPE, and EMR-Lipid dSPE, regarded as green chemistry methods, were evaluated in terms of matrix removal efficiency, recovery rate, and matrix effect. For rapid screening of multi-class target analytes, extracted common ion chromatogram (ECIC) and neutral loss scan (NLS) based on characteristic common MS/MS fragments were performed. To reconfirm, narrow retention time windows, exact mass measurements, and MS/MS spectral matching were utilized to avoid false-positive and -negative results. This study describes a novel method to effectively remove matrix interferences in soft-gels for screening of a wide-range of illegal adulterants, with viewpoint of green chemistry. The developed method was successfully applied to screen and identify 92 illegal adulterants in soft-gel-type supplements.

## 2. Results and Discussion

### 2.1. Extraction and Cleanup Methods

**Figure 1:**
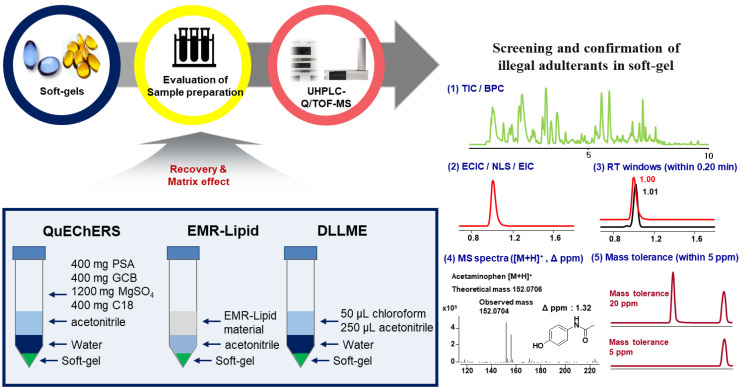
Analytical flow for screening of 92 illegal adulterants in soft-gel type dietary supplements.

The chemical composition of soft-gel formulations consists of lipophilic compounds such as phospholipids, triacylglycerolipids, cholesterol, and sterol esters. Since most fatty acids have both a lipophilic alkyl chain and polar carboxylic acid, it is not easy to selectively extract target adulterants from sample matrix using specific solvent. In the previous study, ethyl acetate was successfully applied to extract illegal adulterants from soft-gel-type supplements [28]. However, using ethyl acetate, a large amount of lipids together with target analytes were co-extracted. When acetonitrile was used, overall extraction efficiency of target illegal adulterants was slightly lower than when using ethyl acetate, but extraction of lipid components was significantly reduced due to low lipid solubility. Thus, in this study, acetonitrile was selected as an extraction solvent, taking into consideration the low matrix effect and reasonable recovery yield. After extraction, the three pretreatment methods of DLLME, QuEChERS-dSPE, and EMR-Lipid dSPE were evaluated in view of the recovery rate and matrix effect (Figure 1).

In DLLME methods, it is necessary to select suitable dispersion and extractant solvents. In previous studies, the influence of several dispersive solvents, such as methanol, acetone, and acetonitrile, was investigated [29,30,31]. Although methanol and acetonitrile as dispersive solvents were shown to have similar extraction efficiencies, taking into consideration matrix co-extraction led to acetonitrile being selected as dispersive solvent for soft-gel samples. Extraction solvents can be divided into two types of organic solvent, namely those with a density lower than water (e.g., 1-undecanol, 2-dodecanol, and 1-octanol) and those of higher density than water (e.g., chloroform and dichloromethane) [32,33]. However, since low density organic solvents including 1-undecanol can bind to matrix interferences, it is difficult to extract illegal adulterants from soft-gel samples [34]. Therefore, in this study, chloroform, which has a high density and concentration factor, was selected as an extraction solvent to improve extraction efficiency.

In QuEChERS-dSPE method to remove lipid components, previous works have reported comparisons of results of extraction efficiencies between dSPE methods, such as primary secondary amine (PSA), graphitized carbon black (GCB), and C18 [23,24,35]. In one previous report, mixed sorbents were used to effectively remove lipid components [35]. Therefore, the same weights of the three sorbents were mixed and added into dSPE kit to remove lipids in dietary supplements more efficiently.

The EMR-Lipid dSPE method is a variation of the QuEChERS-dSPE method applied to effectively remove lipid components using mechanisms of size exclusion and hydrophobic interaction [36]. In this study, the EMR-Lipid kit packed with 500 mg of sorbents was employed.

In this study, since these three extraction and cleanup methods were regarded as green chemistry methodologies, the greenness of analytical procedures was evaluated based on penalty points calculated by Analytical Eco-Scale [37,38]. As shown in Table S1, although all analytical procedures were demonstrated as green chemistry methods, the analytical method including EMR-Lipid dSPE had slightly lower penalty points, compared to QuEChERS-dSPE and DLLME methods.

### 2.2. Comparison of DLLME, QuEChERS-dSPE, and EMR-Lipid dSPE Methods

**Figure 2:**
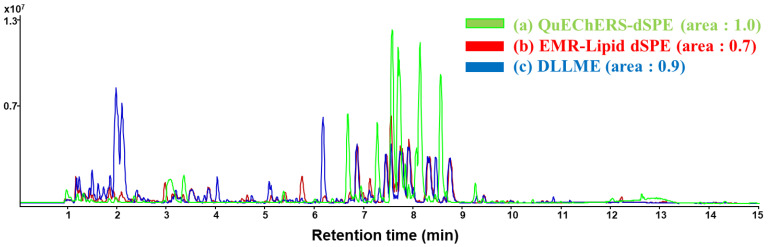
Total ion chromatograms of matrix residues after sample pretreatments of soft-gel by (**a**) QuEChERS-dSPE, (**b**) EMR-Lipid dSPE, and (**c**) DLLME methods.

According to each pretreatment method, total ion chromatograms of uncontaminated soft-gel samples were analyzed by MS scan ranging over *m/z* 100–1100 in positive ion mode using UHPLC-Q/TOF-MS (Figure 2). To compare the matrix removal efficiencies of selected pretreatment methods, the area under the curve (AUC) for each pretreatment method was investigated. When AUC for the QuEChERS-dSPE method was set at 1.0, DLLME and EMR-Lipid dSPE methods were calculated as 0.9 and 0.7, respectively. According to the AUCs for pretreatment methods, the EMR-Lipid dSPE method was shown to be the most effective matrix removal method. Although matrix residues were still present in the sample after EMR-Lipid dSPE, the amount of matrix residues after EMR-Lipid dSPE was considerably reduced compared to other pretreatment methods.

Since matrix residues remained in the samples despite DLLME, QuEChERS-dSPE, and EMR-Lipid dSPE pretreatments, we investigated the influence of matrix residues after respective pretreatments. To evaluate the effects of the co-extracted matrix residues on the qualitative and quantitative analysis of the analytes, recoveries and matrix effects of 92 illegal adulterants spiked in soft-gel samples were investigated according to DLLME, QuEChERS-dSPE, and EMR-Lipid dSPE methods. Overall result values for respective methods are summarized in Table 1.

**Figure 3:**
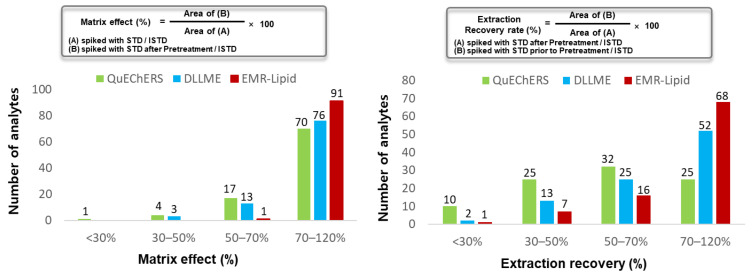
Comparison of matrix effects and extraction recovery for 92 illegal adulterants according to three pretreatment methods.

As shown in Table 1, the recoveries and matrix effects of EMR-Lipid dSPE provided better results compared to those of DLLME and QuEChERS-dSPE. Recovery results for several analytes, however, (such as 4-dimethylaminoantipyrine (47.2%), hongdenafil (53.4%), modafinil (63.3%), olopatadine (39.3%), avanafil (62.3%), betamethasone (58.3%), sulindac (41.2%), and fexofenadine (52.7%)), were contrary to the trend. Moreover, EMR-Lipid dSPE pretreatment provided low recovery rates for several compounds, (such as thioquinapiperifil (16.9%), triprolidine (49.6%), ketorolac (45.3%), levothyroxine (40.0%), and phenylbutazone (32.4%)). Relatively low recovery rates for these compounds might come from their polar characteristics. As shown in Figure 3, 24 compounds of 92 illegal adulterants in EMR-Lipid dSPE showed recoveries below 70%, while 67 and 40 compounds of 92 analytes showed extraction recovery rates below 70% in QuEChERS-dSPE and DLLME, respectively. Furthermore, for the matrix effects of EMR-Lipid method, only one compound of 92 illegal adulterants showed an inappropriate matrix effect of 69.8%, while for QuEChERS and DLLME, 22 and 16 compounds showed inappropriate matrix effects below 70%. Despite poor extraction efficiencies for several compounds in EMR-Lipid dSPE pretreatment, [M+H]^+^ ions for 92 illegal adulterants were successfully detected at 5 ppm levels in positive ion mode and effects on qualitative analysis of all analytes were negligible.

In DLLME, it is difficult to select suitable dispersion and extractant solvents to effectively extract a wide range of illegal adulterants in soft-gel samples. In QuEChERS-dSPE, lipid components in soft-gels were cleaned up through hydrophobic and Lewis acid–base interactions with dispersive sorbents [39]. However, since these chemical interactions were not selective, the QuEChERS-dSPE method could not effectively control cleanup for lipids in soft-gel samples and provide extraction for all target adulterants. EMR-Lipid dSPE method is a modified QuEChERS-dSPE method that adds size exclusion separation mechanism to a conventional QuEChERS-dSPE method [36]. In EMR-Lipid dSPE, while lipids and lipid-like molecules are selectively bound to sorbents, most chemical adulterants dissimilar to lipid structures cannot bind to EMR-Lipid dSPE sorbents. The EMR-Lipid dSPE method could selectively extract illegal adulterants in soft-gel-type dietary supplements without the co-extraction of matrix lipids.

### 2.3. Analysis of 92 Illegal Adulterants by UHPLC-Q/TOF-MS

**Figure 4:**
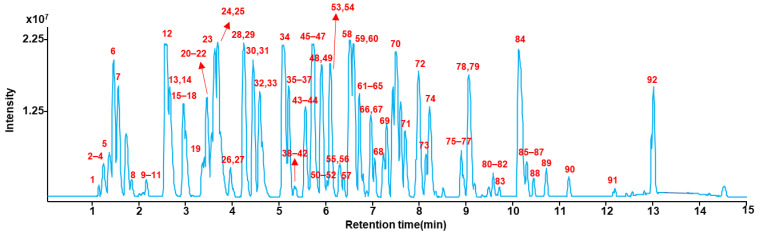
Total ion chromatogram of 92 illegal adulterants under optimized LC-MS conditions in positive ion mode.

In this study, a UHPLC-Q/TOF-MS method was developed to determine multi-class 92 illegal adulterants. Conditions for LC separation and MS detection were modified slightly based on our previous studies [16,17]. To efficiently separate 92 illegal adulterants, several LC conditions (such as three analytical columns (HSS T3, UPLC BEH C18, and biphenyl with same dimension (150 × 2.1 mm, 1.7 μm)), mobile phase flow rates (250, 300, 350, and 400 μL/min), and column temperatures (20, 30, 40, and 50 °C)) were evaluated. As shown in Figure 4, optimal chromatographic separations of 92 multi class analytes were achieved under the BEH C18 column at 40 °C with flow rate at 300 μL/min. Although efficient LC separations of overall analytes were obtained under optimized LC conditions, several peaks of 92 illegal adulterants were overlapped. Most co-eluted adulterants provided different [M+H]^+^ ions, except for hongdenafil and dimethylacetildenafil, which are isobaric compounds (*m/z* 467.2765) eluted within the same retention time window (3.43 and 3.54 min). Nevertheless, since they provided characteristic MS/MS fragment patterns from piperazine ring moieties in the chemical structure, they could be successfully separated. All analytes were sensitively detected under optimized MS conditions described in Section 3.5. Based on optimized UHPLC-Q/TOF-MS conditions, obtained retention times and mass errors of 92 illegal adulterants are summarized in Table S2.

**Figure 5:**
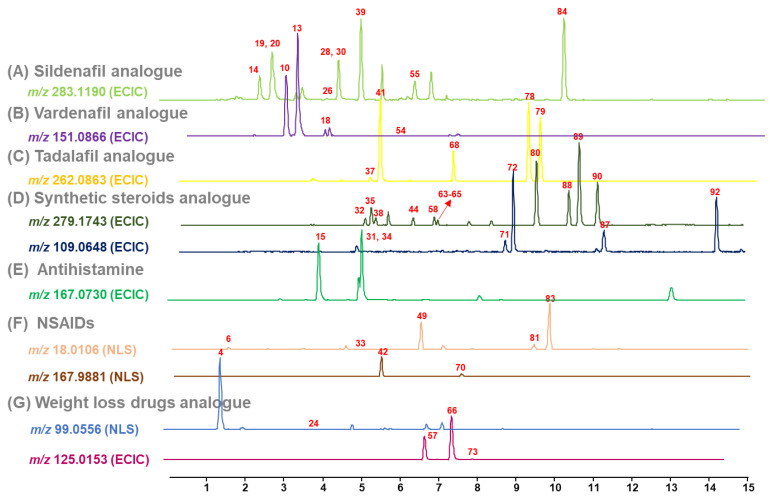
ECICs and NLSs of illegal adulterants after application of EMR-Lipid dSPE pretreatment to soft-gel samples spiked with analytes at 2 μg/g level.

It was reported that ECIC and NLS using common fragments enabled rapid screening of various illegal adulterants in dietary supplements [40]. In this study, we investigated common MS/MS fragment ions and neutral loss molecules of 92 illegal adulterants based on previous studies [5,16,17,28]. As shown in Figure 5, seven ECICs for erectile dysfunction drugs, synthetic steroids, antihistamines, and weight-loss drugs and three NLSs for NSAIDs and weight-loss drugs were constructed to screen multi-class illegal adulterants in soft-gel samples. Although 49 of 92 analytes were screened by seven ECICs and three NLSs, all illegal adulterants could be screened by individual EIC. Therefore, a UHPLC-Q/TOF-MS method combined with EMR-Lipid dSPE pretreatment provided sufficient detection sensitivity to screen and identify all target adulterants in soft-gel samples.

### 2.4. Method Applications

The developed analytical method to screen 92 illegal adulterants in soft-gel samples was validated in terms of linearity, detection limits, precision and accuracy, recovery rates, and matrix effects. Overall validation results were summarized in Table 1 and Table 2. In this study, calculated LODs for 92 targeted compounds were comparable with previously reported analytical methods using UHPLC-high resolution MS [41,42,43]. The previously reported methods provided LOD ranges within 0.3–2 ng/g, 0.01–0.04 μg/g, and 0.12–1.50 μg/mL, for 13 weight-loss drugs, 50 antihypertensive adulterants, and 20 anti-gout and anti-osteoporosis drugs, respectively. Under the optimized conditions, LODs ranged from 0.1 to 16 ng/g. Furthermore, most substances adulterated in illegal drugs and supplements were found at mg/g levels [41,42,43,44]. Considering the concentration levels of detected adulterants in previous studies, this method enabled sensitive detection for screening of 92 illegal adulterants. Table 2 shows precision and accuracy results for 92 illegal adulterants. Overall precision and accuracy were within 0.09–14.65% and 81.6–116.6%, respectively. To evaluate the established method, 10 soft-gel-type samples randomly collected from internet and domestic markets were applied using this method. The EMR-Lipid dSPE method was employed to extract 92 illegal adulterants and remove lipids from soft-gel matrices. After EMR-Lipid dSPE, constructed EIC, ECIC, and NLS using exact mass and MS/MS fragments were utilized to rapidly screen multi-class illegal adulterants. To prevent false positive/negative results for screening of illegal adulterants in soft-gel-type sample matrices, suspected compounds were confirmed using a home-built library that included retention time, exact mass, and MS/MS spectra.

**Figure 6:**
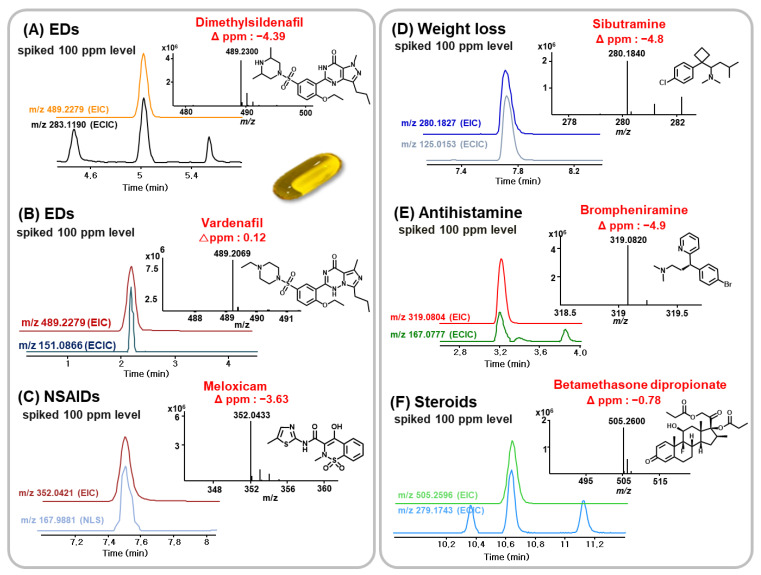
EIC, ECIC and NLS screening and confirmation of illegal adulterants in soft-gel samples.

In this study, since no illegal adulterants were determined in collected soft-gel samples, target adulterants were deliberately added to the soft-gels. Using UHPLC-Q/TOF-MS combined with EMR-Lipid dSPE pretreatment, 92 illegal adulterants were successfully determined. As shown in Figure 5, seven ECICs and three NLSs using common fragments were sufficient to screen various illegal adulterants in soft-gel samples at 2 μg/g. In particular, the EIC, ECIC, and NLS screening methods combined with exact mass measurements (±5 ppm) enabled the rapid and accurate identification of multi-class illegal adulterants in soft-gel samples (Figure 6).

## 3. Experiments

### 3.1. Chemicals and Materials

The chemical structures of the 92 illegal adulterant (25 erectile dysfunction drugs, 15 steroids, 7 anabolic steroids, 12 antihistamine, 12 NSAIDS, 4 diuretics, and 17 weight loss drugs) standards and 10 deuterium labelled internal standards are depicted in Figures S1–S5. Reference standards of the 92 illegal adulterants were obtained from Sigma Aldrich (St. Louis, MO, USA), Toronto Research Chemicals Inc. (Toronto, ON, Canada), TLC Pharmaceutical Standards (Vaughan, ON, Canada), and Steraloids (Newport, RI, USA). The 92 targeted substances were selected as possible illegal adulterants used in dietary supplements and counterfeits distributed in Korean markets. Sildenafil-d_8_ (purity 99.9%), vardenafil-d_5_ (purity 98.5%), tadalafil-d_5_ (purity 99.9%) were purchased from TLC Pharmachem (Vaughan, ON, Canada), testosterone-d_3_ (purity 99.9%), progesterone-d_9_ (purity 99.9%), promethazine-d_4_ (purity 99.9%) were obtained from Sigma Aldrich (St. Louis, MO, USA), and tolbutamide-d_9_ (purity 99.9%), metformin-d_6_ (purity 99.9%), acetaminophen-d_4_ (purity 99.9%) were bought from Toronto Research Chemicals Inc. as isotope-labeled internal standards. The soft-gel-type supplements free from adulterants were used as blank samples. All reagents and organic solvents were analytical grades or better. Methanol (MeOH), acetonitrile (ACN), and formic acid were purchased from Honeywell (Morris Plains, NJ, USA). Acetonitrile was filtered through a 0.45-μm membrane filter and degassed for 10 min. In addition, de-ionized water (DW) was produced using a Millipore Direct-Q3 purification system from the Millipore Corporation (Billerica, MA, USA), filtered through a 0.2-μm membrane filter, and degassed for 10 min prior to use.

### 3.2. Preparation of Reference Standards

Individual standards were dissolved in methanol at 1000 μg/mL. Each stock solution was kept in an amber vial and was vortex mixed for 30 s. Deuterated internal standard solutions (sildenafil-d_8_, vardenafil-d_5,_ tadalafil-d_3_, testosterone-d_3_, progesterone-d_9_, promethazine-d_4_, tolbutamide-d_9_, metformin-d_6_, acetaminophen-d_4_) were prepared in methanol at a 100 μg/mL concentration level. The working solutions of all the compounds were prepared by successively diluting stock solutions. The standard stock and working solutions were stored at −20 °C.

### 3.3. Sample Preparation

Herbal medicines and dietary supplements are typically in the form of soft-gels. Soft-gel samples were opened, and the contents removed prior to homogenization. Analytes were extracted from sample by three protocols, QuEChERS-dSPE, EMR-lipid dSPE, and dispersive liquid-liquid microextraction (DLLME), for evaluation of sample cleanup. The homogenized test portions (1.00 ± 0.01 g) were taken and then the extraction of illegal adulterants was performed.

For the DLLME procedure, 1 g of soft-gel samples was added to 5 mL acetonitrile in a 50-mL polypropylene (PP) centrifuge tube, followed by sonication for 10 min. The sample was centrifuged at 4000 rpm for 10 min and the supernatant was transferred into another 50-mL PP tube. The acetonitrile extract was evaporated under nitrogen gas and then added to 5 mL of de-ionized water. For extraction, a mixture of 50 μL of chloroform (extraction solvent) and 250 μL of acetonitrile (dispersive solvent) was rapidly injected into the working solution using a 1-mL Hamilton syringe. The tube was shaken for 3 min, until a cloudy solution formed, and centrifugation followed at 4000 rpm for 10 min. The chloroform droplets were transferred to a 1.5-mL vial and were evaporated under nitrogen gas, then re-dissolved with 50 μL of initial mobile phase.

For the QuEChERS-dSPE procedure, 1 g of soft-gel samples was added to 10 mL of 2% (*v*/*v*) formic acid in de-ionized water and 10 mL acetonitrile into a 50-mL PP centrifuge tube, followed by sonication for 10 min. In the next step, 4 g of anhydrous MgSO_4_ and 1 g of NaCl were added to the tube. The sample was vortex mixed for 3 min followed by centrifugation at 4000 rpm for 10 min. The supernatant was transferred into a 50-mL PP tube, which already contained the Agilent Bond Elut AOAC extraction kit (5982-5456, 400 mg of PSA, 400 mg of GCB, 1200 mg of MgSO_4_, 400 mg of C18), shaken for 3 min, and centrifuged at 4000 rpm for 10 min. The acetonitrile extracts were evaporated under nitrogen gas and then re-dissolved with 50 μL of initial mobile phase.

For the EMR-Lipid dSPE procedure, 1 g of soft-gel samples was added to 5 mL acetonitrile in a 50-mL PP centrifuge tube, followed by sonication for 10 min. In the next step, EMR-Lipid material kit (5982-1010, Agilent Bond Elut QuEChERS Dispersive kit) was activated by 5 mL of de-ionized water prior to use, transferred to the acetonitrile extract, and the tube was shaken for 3 min followed by centrifugation at 4000 rpm for 10 min. The supernatant was transferred into a 50-mL PP tube and another EMR-Lipid kit (5982-0101, Agilent Bond Elut QuEChERS—NaCl/MgSO_4,_ (1/4, *w*/*w*) anhydrous) was added to the same tube. The tube was shaken for 3 min followed by centrifugation at 4000 rpm for 10 min. The acetonitrile extract was evaporated under nitrogen gas and then re-dissolved with 50 μL of initial LC mobile phase. The three above mentioned sample pretreatment procedures are depicted in Figure S6.

### 3.4. UHPLC Conditions

Chromatographic separations were performed on Agilent 1290 UHPLC system (Agilent Technologies, Santa Clara, CA, USA). The chromatographic separation was carried out on Waters ACQUITY^®^ UPLC BEH C18 column (150 × 2.1 mm, i.d., 1.7 μm). Mobile phase A and B were 0.1% (*v*/*v*) formic acid in water and acetonitrile, respectively. Gradient elution was initiated with 30% of mobile phase B for 0.0–1.0 min, 30%–41% of B for 1.0–4.0 min, 41%–75% of B for 4.0–11.0 min, 75%–80% of B for 11.0–11.1 min, 80%–100% of B for 11.1–13.0 min, and 100% B for 13.0–15.0 min. The flow rate, injection volume, and column temperature were set at 300 μL/min, 2 μL, and 40 °C respectively.

### 3.5. Q/TOF-MS Conditions

All LC–MS and LC-MS/MS experiments were performed using a 6530 accurate mass quadrupole time-of-flight mass spectrometer instrument (Agilent Technologies, Santa Clara, CA, USA). This instrument was operated in extended dynamic range of 2 GHz (*m/z* 3200 Th) in the high-resolution mode. Positive ions of analytes are generated using an ESI source with conditions as follows: the super-heated nitrogen sheath gas temperature, 350 ℃; and flow, 11 L/min. Mass spectrometer conditions were set to the followings: capillary voltage (Vcap), 4000 V; nebulizer pressure, 45 psi, drying gas, 8 L/min; and gas temperature, 300 ℃. The fragmentor, skimmer, and octapole RF voltages were set to be 175, 65, and 750 V, respectively. The mass scan range was *m/z* 100–1100, and reference masses of *m/z* 121.050873 (purine) and 922.009798 (HP-0921) were used to calibrate the mass axis during analysis. The exact mass measurements for all of the MS data were controlled by MassHunter software B.02.00 ChemStation (Agilent Technologies, Santa Clara, CA, USA). The MS/MS parameters are as follow: fragmentor, 300 V; skimmer, 65 V; and octapole RF, 750 V. The MS/MS experiments were performed at a fixed collision energy of 30 V. For MS/MS spectra of individual analytes, the [M+H]^+^ ions were selected as precursor ions.

### 3.6. Method Validation

Analytical performance of the developed analytical method was validated with linearity (r^2^), limits of detection (LODs), precision and accuracy, recovery rates, and matrix effects, based on ICH Q2 guideline [45]. Each calibration curve was constructed by plotting the peak area ratio of each analyte to the corresponding internal standards. The linearity was determined from constructed calibration curve for each analyte with different concentration ranges. The LODs were defined as the lowest concentration levels yielding a signal-to-noise ratio at 3. To support the analysis of illegal adulterants in dietary supplements, linear dynamic ranges and LODs were calculated based on 1 g of soft-gel homogenates. Precision and accuracy were evaluated in triplicate using blank samples spiked with standards at 50 ng/g for 51 analytes and 200 ng/g for 41 analytes. Further, intra- and inter-day precision results were determined within a day and three consecutive days, respectively. Recovery rates were calculated by spiking with standard mixture solution before and after sample extraction. The internal standards were added into the reconstituted sample. The recovery rates were calculated as follows: recovery rates (%) = A/B × 100, where A and B are the peak area ratio of spiked analytes to internal standard before and after sample extraction, respectively. Matrix effects were evaluated by calculating of following equation: matrix effects (%) = B/C × 100, where C is the peak area ratio of analytes to internal standard in standard solution.

### 3.7. Application

Ten soft-gel-type dietary supplements obtained from internet and domestic markets were analyzed using the developed method. The suitability of the sample pretreatment method and screening method was evaluated by analyzing supplement samples intentionally spiked with drugs. Confirmation of analytes was accomplished on the basis of matching the retention times, accurate mass values, and MS/MS spectra with their corresponding reference library database. For confirmation of analytes, mass tolerance and retention time deviation were required to be within ±5 ppm and ±0.20 min, respectively.

## 4. Conclusions

A UHPLC-Q/TOF-MS method combined with EMR-Lipid dSPE was developed and validated to screen and confirm 92 illegal adulterants in soft-gel-type dietary supplements. The developed analytical procedures were regarded as a green chemistry method with relatively low penalty points. Furthermore, EMR-Lipid dSPE provided not only better extraction recovery rates, but also better matrix cleanup efficiencies. Therefore, compared to soft-gel extracts using DLLME or QuEChERS-dSPE, sample extracts purified with EMR-Lipid dSPE pretreatment could decrease the potential risk for false-positive and -negative results coming from matrix residues. The ECIC and NLS method enabled a rapid and simple screening of multi-class illegal adulterants, since seven ECICs and three NLSs were constructed using MS/MS common fragments based on characteristic moieties of illegal adulterants. Furthermore, based on homemade library, a reconfirmation method including narrow retention time windows (±0.2 min), exact mass measurements (±5 ppm), and MS/MS spectral matching could reinforce screening results of illegal adulterants in soft-gel-type samples. To evaluate the developed method, this analytical method was applied to 10 soft-gel-type dietary supplements randomly collected from internet and domestic markets. The established method was verified to provide effective cleanup of lipids and lipid-like interferences from soft-gels, as well as extraction of illegal adulterants. Consequentially, this study provides a promising tool for the screening and confirmation of multi-class illegal adulterants in soft-gel-type dietary supplements and will be helpful to develop more environmentally friendly routine analytical methods for identifying dietary supplements illegally adulterated with various drugs.

## Figures and Tables

**Table 1:** Recovery rates (%) and matrix effects (%) of 92 adulterants in soft-gel samples at 2 μg/g level (*n* = 3) by three pretreatment methods.

No.	Analyte	QuEChERS		EMR-Lipid		DLLME	
		R ^1^ (RSD ^3^)%	M ^2^ (RSD ^3^)%	R ^1^ (RSD ^3^)%	M ^2^ (RSD ^3^)%	R ^1^ (RSD ^3^)%	M ^2^ (RSD ^3^)%
1	Metformin	82.6 (3.2)	83.6 (5.5)	90.4 (6.8)	86.6 (5.1)	62.4 (7.5)	70.8 (14.9)
2	Amiloride HCl	81.3 (5.3)	63.7 (2.0)	101.5 (1.8)	84.8 (9.0)	79.4 (3.2)	113.4 (6.4)
3	4-Dimethylaminoantipyrine	47.8 (9.3)	60.6 (1.7)	47.2 (10.9)	81.3 (1.5)	40.8 (4.8)	42.5 (9.5)
4	Theophylline	38.1 (2.9)	44.1 (7.1)	91.4 (12.4)	93.2 (5.7)	72.8 (6.7)	75.0 (7.7)
5	Ephedrine HCl	77.3 (7.7)	71.8 (3.7)	104.1 (6.7)	103.7 (14.3)	92.0 (3.5)	102.1 (6.9)
6	Acetaminophen	75.1 (6.2)	53.3 (8.8)	119.4 (7.7)	81.0 (10.3)	78.1 (3.6)	97.1 (5.1)
7	Triamterene	37.0 (4.4)	25.1 (12.3)	99.8 (5.9)	96.0 (7.4)	53.1 (8.7)	54.7 (4.4)
8	Captopril	37.2 (3.3)	42.6 (11.5)	69.2 (8.9)	99.0 (2.4)	25.4 (7.4)	38.0 (3.9)
9	Yohimbin	75.1 (8.9)	75.6 (5.9)	78.0 (3.2)	88.9 (2.9)	67.7 (3.1)	81.9 (3.8)
10	Hydroxyvardenafil	33.6 (7.1)	79.2 (5.2)	67.0 (5.6)	91.1 (6.3)	63.8 (7.1)	80.1 (0.4)
11	Thioquinapiperifil	14.5 (7.8)	48.3 (5.3)	16.9 (8.1)	95.6 (2.7)	14.3 (4.6)	57.0 (8.9)
12	Bambuterol	62.3 (6.7)	91.9 (4.1)	66.6 (9.2)	91.1 (4.9)	58.8 (9.4)	82.3 (5.6)
13	Vardenafil	29.0 (4.9)	65.2 (3.9)	77.6 (9.0)	90.3 (7.5)	70.2 (3.4)	78.9 (7.4)
14	Carbodenafil	48.8 (9.1)	83.1 (7.8)	66.8 (8.4)	87.2 (1.5)	57.1 (9.2)	54.3 (13.1)
15	Brompheniramine	26.8 (5.3)	76.5 (3.0)	50.8 (12.8)	78.5 (11.9)	45.8 (6.7)	68.8 (2.3)
16	Bupropion HCl	92.3 (6.1)	84.3 (1.8)	97.2 (7.7)	82.4 (2.1)	71.6 (5.6)	86.2 (2.2)
17	Triprolidine	24.3 (8.2)	94.2 (4.9)	49.6 (8.8)	69.8 (11.2)	33.4 (5.4)	91.6 (6.7)
18	Norneovardenafil	8.7 (12.9)	56.5 (6.9)	71.8 (2.1)	86.4 (8.3)	67.3 (4.9)	103.0 (9.8)
19	Hongdenafil	12.1 (9.2)	92.2 (3.1)	53.4 (9.0)	109.5 (9.7)	61.2 (3.8)	74.8 (4.3)
20	Dimethylacetildenafil	11.9 (4.7)	82.4 (4.6)	106.4 (4.7)	84.7 (9.7)	88.2 (2.4)	95.1 (7.0)
21	Ketotifen fumarate salt	74.8 (7.7)	94.6 (12.6)	98.9 (6.4)	90.9 (10.2)	76.4 (6.8)	87.9 (4.3)
22	Icariin	46.7 (2.5)	69.6 (8.4)	61.3 (2.1)	78.1 (5.9)	60.8 (7.6)	85.2 (2.0)
23	Astemizole	70.8 (9.1)	103.6 (4.7)	93.8 (9.6)	114.8 (9.9)	86.0 (6.2)	106.2 (3.2)
24	Propranolol	78.6 (6.4)	83.6 (1.8)	93.5 (1.3)	78.2 (2.4)	72.8 (6.5)	87.9 (1.2)
25	Modafinil	52.4 (2.7)	49.8 (6.0)	63.3 (12.2)	73.8 (9.3)	64.4 (9.9)	70.1 (6.4)
26	Oxohongdenafil	38.4 (7.9)	109.2 (2.9)	69.4 (6.2)	96.1 (5.9)	42.7 (9.5)	102.5 (4.9)
27	Olopatadine	44.2 (4.4)	74.4 (5.2)	39.3 (5.1)	84.4 (10.6)	77.8 (9.4)	80.8 (0.8)
28	Sildenafil	40.9 (4.8)	91.4 (0.9)	103.1 (6.9)	99.2 (4.4)	82.9 (4.1)	84.7 (6.4)
29	Avanafil	35.7 (3.2)	79.6 (4.4)	62.3 (12.2)	70.2 (12.4)	72.9 (5.6)	86.7 (2.9)
30	Dimethylsildenafil	59.7 (2.1)	82.0 (2.6)	87.8 (3.2)	93.3 (1.2)	86.2 (4.1)	86.5 (4.6)
31	Diphenhydramine	84.8 (2.3)	73.7 (6.3)	99.0 (5.8)	84.9 (5.5)	75.4 (5.2)	77.9 (7.4)
32	Methylprednisolone	47.0 (4.8)	89.1 (4.9)	82.7 (4.4)	88.6 (2.5)	80.6 (7.7)	84.0 (2.9)
33	Carbamazepine	90.4 (9.3)	91.3 (1.4)	94.5 (1.8)	96.7 (4.4)	80.1 (3.3)	84.4 (1.4)
34	Dimenhydrinate	69.2 (8.1)	72.8 (4.8)	102.6 (1.5)	95.5 (2.2)	82.7 (6.7)	84.4 (2.8)
35	Betamethasone	35.8 (9.7)	74.3 (0.6)	58.3 (4.3)	72.1 (4.1)	59.7 (9.6)	76.8 (3.2)
36	Eplerenone	67.3 (6.3)	55.1 (2.6)	95.3 (3.8)	94.5 (3.0)	74.8 (8.3)	69.0 (4.6)
37	Acetaminotadalafil	47.8 (2.6)	66.0 (8.4)	97.5 (5.4)	98.8 (4.3)	43.1 (4.5)	53.5 (8.6)
38	Dexamethasone	58.6 (5.6)	82.2 (14.8)	93.0 (10.8)	92.6 (2.6)	37.7 (4.4)	51.8 (8.5)
39	Udenafil	57.5 (2.2)	93.3 (3.0)	110.9 (7.3)	104.0 (4.0)	46.8 (6.8)	79.3 (8.7)
40	Promethazine	40.2 (6.8)	58.2 (7.0)	100.3 (0.7)	99.9 (5.5)	69.7 (8.1)	62.2 (4.9)
41	Demethyltadalafil	70.8 (3.3)	89.2 (14.0)	97.8 (4.3)	93.0 (0.9)	66.8 (9.4)	106.4 (6.7)
42	Piroxicam	79.1 (5.5)	108.7 (2.9)	101.6 (6.3)	100.7 (6.4)	83.2 (3.1)	104.2 (8.4)
43	Paroxetine	59.8 (6.4)	74.4 (8.8)	104.0 (8.5)	101.6 (12.1)	48.1 (5.4)	53.6 (10.4)
44	Beclomethasone	41.8 (2.5)	100.2 (13.8)	108.0 (5.3)	98.6 (4.6)	79.2 (4.6)	69.5 (6.3)
45	4-Isopropylantipyrine	81.4 (7.7)	112.9 (4.0)	97.4 (11.3)	87.9 (5.7)	86.0 (7.6)	114.3 (3.1)
46	Phenolphthalein	45.2 (6.7)	106.8 (9.0)	99.8 (9.9)	88.8 (6.8)	82.9 (4.9)	98.0 (2.5)
47	Ketorolac	34.7 (9.6)	80.8 (4.9)	45.3 (5.5)	73.4 (7.9)	38.6 (3.2)	57.1 (3.6)
48	Flunisolide	65.4 (7.8)	55.1 (7.7)	113.2 (6.7)	88.6 (2.9)	80.2 (3.9)	85.4 (4.2)
49	Sulindac	59.3 (5.1)	81.0 (2.4)	41.2 (9.9)	71.8 (6.4)	75.4 (4.8)	103.4 (9.3)
50	Cyproheptadine	50.7 (9.1)	84.3 (1.8)	91.7 (11.4)	95.6 (4.2)	77.9 (3.8)	86.6 (2.4)
51	Levothyroxine	39.2 (6.5)	76.7 (4.6)	40.0 (5.8)	82.8 (6.3)	37.8 (6.6)	83.3 (1.4)
52	Bisacodyl	86.5 (8.2)	87.8 (3.6)	102.5 (0.4)	97.6 (1.7)	89.1 (4.4)	92.7 (3.1)
53	Boldenone	49.8 (5.1)	76.6 (5.2)	86.2 (8.2)	84.9 (3.0)	67.3 (8.8)	80.0 (10.7)
54	Desulfovardenafil	38.7 (3.5)	85.4 (8.8)	96.2 (9.2)	96.0 (4.7)	77.3 (3.2)	88.8 (11.1)
55	Benzylsildenafil	86.4 (9.6)	93.4 (11.3)	97.7 (6.9)	94.2 (1.3)	70.1 (7.6)	73.8 (23.1)
56	Xanthoanthrafil	88.7 (9.3)	82.6 (11.2)	103.5 (10.5)	100.4 (5.6)	73.4 (7.4)	88.1 (8.2)
57	Didesmethylsibutramine	79.2 (6.7)	84.5 (4.5)	114.3 (4.2)	98.5 (7.6)	80.3 (6.2)	85.2 (4.7)
58	Prednisone-21-acetate	59.2 (1.9)	104.9 (1.4)	103.4 (2.3)	101.8 (4.3)	75.6 (5.3)	108.4 (6.6)
59	Fexofenadine	72.6 (8.1)	86.3 (2.5)	52.7 (5.3)	79.3 (2.4)	47.8 (5.9)	72.2 (5.4)
60	Fluoxetine HCl	75.5 (3.5)	82.0 (7.9)	96.9 (7.1)	88.7 (4.7)	76.3 (7.1)	86.8 (2.9)
61	Dapoxetine	24.8 (6.3)	89.9 (7.6)	91.9 (5.6)	100.8 (1.6)	76.4 (5.8)	85.5 (1.6)
62	Mirodenafil	83.3 (3.4)	94.7 (9.5)	105.5 (7.6)	102.3 (10.4)	91.7 (2.9)	114.6 (7.3)
63	Prednisolone-21-acetate	61.2 (9.2)	82.4 (1.3)	108.4 (4.3)	119.2 (7.2)	89.6 (3.5)	102.2 (8.9)
64	Beclomethasone-21-hemisuccinate	58.1 (5.9)	75.8 (5.7)	61.5 (2.8)	89.2 (1.4)	53.6 (5.1)	79.7 (5.4)
65	Cortisone-21-acetate	64.0 (9.4)	75.3 (11.0)	107.0 (7.9)	109.4 (3.8)	75.4 (8.1)	77.8 (11.9)
66	Sibutramine	68.6 (3.6)	81.4 (10.8)	89.6 (5.1)	94.1 (8.3)	69.4 (3.2)	89.0 (4.4)
67	Sertraline HCl	57.5 (8.8)	75.1 (3.1)	101.3 (1.7)	113.7 (9.7)	52.8 (10.2)	49.7 (5.1)
68	Homotadalafil	60.8 (6.9)	95.5 (3.2)	93.2 (1.7)	89.8 (9.2)	66.9 (5.7)	89.2 (9.8)
69	Boldione	67.3 (3.2)	73.2 (1.3)	97.7 (13.3)	91.2 (1.5)	79.3 (7.8)	84.4 (5.8)
70	Meloxicam	78.3 (4.8)	84.0 (5.7)	90.4 (6.8)	92.8 (6.3)	70.1 (6.2)	75.6 (1.3)
71	Mibolerone	51.5 (4.1)	62.2 (1.6)	100.1 (5.0)	101.7 (2.8)	73.8 (5.9)	83.5 (3.9)
72	Danazol (M)	34.8 (6.8)	61.7 (4.6)	85.4 (5.7)	83.5 (6.0)	56.6 (5.4)	69.5 (14.6)
73	Chlorosibutramine	55.4 (4.6)	65.8 (5.5)	89.4 (1.4)	91.2 (3.2)	74.0 (4.8)	91.7 (3.5)
74	Spironolactone	74.1 (2.4)	76.5 (2.5)	82.8 (17.2)	88.4 (8.1)	78.3 (6.2)	80.4 (1.3)
75	Fluocinonide	65.6 (5.2)	77.2 (3.0)	107.3 (6.1)	114.2 (4.2)	75.4 (4.3)	84.3 (0.5)
76	Calusterone	65.3 (8.1)	69.0 (4.1)	117.0 (0.7)	109.3 (9.1)	59.3 (3.6)	80.7 (7.3)
77	Clostebol	72.9 (5.2)	83.9 (3.9)	96.9 (8.0)	100.5 (2.4)	74.1 (5.4)	85.7 (2.0)
78	Cyclopentyltadalafil	43.2 (7.9)	65.2 (3.3)	101.3 (8.3)	112.2 (7.1)	64.6 (5.1)	80.0 (10.3)
79	Chloropretadalafil	42.9 (7.8)	64.2 (9.6)	95.0 (1.3)	97.3 (3.9)	64.4 (9.5)	70.2 (8.9)
80	Betamethasone-17-valerate	34.8 (8.3)	71.2 (8.6)	105.6 (9.0)	94.2 (7.9)	68.3 (8.6)	53.5 (9.8)
81	Diclofenac	66.8 (3.7)	63.6 (2.8)	67.7 (3.6)	87.5 (5.1)	60.2 (5.1)	75.9 (6.2)
82	Indomethacin	52.3 (2.9)	77.7 (9.7)	58.1 (4.2)	95.4 (2.6)	43.1 (6.7)	79.9 (4.7)
83	Aceclofenac	27.1 (6.3)	71.3 (3.6)	61.5 (6.6)	91.8 (1.2)	60.5 (3.9)	76.5 (1.6)
84	Imidazosagatriazinone	64.2 (8.5)	104.3 (7.5)	97.0 (0.9)	96.5 (3.7)	71.6 (3.2)	102.7 (4.3)
85	Terfenadine	76.1 (9.0)	86.3 (3.6)	94.4 (11.2)	95.4 (5.4)	80.1 (4.2)	82.2 (10.6)
86	Phenylbutazone	25.3 (7.1)	98.3 (8.5)	32.4 (9.2)	94.4 (6.2)	30.1 (7.8)	93.7 (4.9)
87	Norbolethone	54.7 (3.8)	76.8 (3.5)	106.0 (5.1)	107.5 (8.8)	75.9 (6.4)	77.3 (3.2)
88	Betamethasone-21-valerate	64.9 (8.5)	71.6 (12.7)	102.1 (2.2)	109.4 (6.9)	74.1 (6.2)	82.7 (5.1)
89	Betamethasone dipropionate	57.2 (2.8)	73.9 (1.6)	94.6 (5.3)	95.5 (4.3)	73.5 (4.5)	84.4 (1.8)
90	Beclomethasone dipropionate	64.6 (7.6)	73.9 (1.6)	95.7 (7.4)	97.1 (6.7)	73.2 (7.1)	84.9 (2.1)
91	Rimonabant	63.8 (4.3)	74.3 (2.0)	108.3 (7.1)	107.5 (9.2)	78.8 (5.2)	88.2 (13.2)
92	Testosterone-17-propionate	69.7 (4.9)	72.1 (9.1)	98.5 (6.0)	99.2 (3.1)	76.7 (4.4)	92.7 (1.4)

^1^ R: recovery rate, ^2^ M: matrix effect, ^3^ RSD: relative standard deviation.

**Table 2:** Method validation results of 92 illegal adulterants obtained by UHPLC-Q/TOF-MS.

No.	Analyte	Linearity Range (ng/g)	Correlation Coefficient (r^2^)	LOD (ng/g)	Precision (%)		Accuracy (RSD) (%)
					Intra-Day	Inter-Day	
1	Metformin ^1^	25–500	0.9924	4	0.09	12.14	101.1 (3.6)
2	Amiloride HCl ^1^	25–500	0.9911	15	2.22	10.34	87.4 (9.4)
3	4-Dimethylaminoantipyrine ^1^	25–2000	0.9903	0.7	0.97	1.35	101.5 (3.7)
4	Theophylline ^1^	25–2000	0.9909	2	0.12	3.89	115.5 (0.1)
5	Ephedrine HCl ^1^	25–1000	0.9938	0.3	0.44	0.34	100.7 (0.5)
6	Acetaminophen ^1^	25–2000	0.9934	2	1.02	9.46	98.5 (0.6)
7	Triamterene ^1^	25–500	0.9943	0.4	1.98	12.23	110.2 (7.4)
8	Captopril ^1^	25–2000	0.9956	11	3.41	5.27	95.7 (3.0)
9	Yohimbin ^1^	5–500	0.9979	7	5.02	5.16	97.2 (8.4)
10	Hydroxyvardenafil ^1^	25–500	0.9991	4	4.35	0.27	95.6 (0.2)
11	Thioquinapiperifil ^2^	5–500	0.9967	2	4.07	2.54	103.0 (1.5)
12	Bambuterol ^2^	1–500	0.9962	0.2	3.39	0.62	115.7 (4.5)
13	Vardenafil ^2^	1–500	0.9978	0.4	2.86	2.12	86.7 (0.5)
14	Carbodenafil ^2^	5–500	0.9972	1	1.12	5.42	103.9 (0.4)
15	Brompheniramine ^2^	1–500	0.9966	0.1	3.31	0.56	98.7 (1.8)
16	Bupropion HCl ^1^	25–500	0.9935	0.4	5.32	6.72	105.9 (3.1)
17	Triprolidine ^2^	1–500	0.9979	0.3	6.02	1.81	112.5 (4.7)
18	Norneovardenafil ^2^	1–500	0.9983	0.1	3.50	3.40	116.6 (1.5)
19	Hongdenafil ^2^	1–500	0.9971	0.5	6.29	5.42	116.0 (1.5)
20	Dimethylacetildenafil ^2^	1–500	0.9985	0.5	5.74	5.21	92.9 (1.8)
21	Ketotifen fumarate salt ^2^	1–500	0.9975	0.1	6.69	1.02	100.8 (2.6)
22	Icariin ^1^	25–500	0.9982	8	2.05	0.27	110.8 (1.9)
23	Astemizole ^2^	5–500	0.9961	2	0.16	1.45	88.5 (5.2)
24	Propranolol ^1^	25–500	0.9915	0.1	1.16	14.18	96.8 (2.4)
25	Modafinil ^1^	25–1000	0.9961	0.5	1.45	12.58	106.0 (4.2)
26	Oxohongdenafil ^1^	25–500	0.9971	4	1.73	3.83	115.4 (1.2)
27	Olopatadine ^2^	1–500	0.9973	0.4	4.31	0.81	91.8 (1.7)
28	Sildenafil ^2^	5–500	0.9969	2	4.02	4.03	95.6 (9.2)
29	Avanafil ^2^	1–500	0.9997	0.5	6.29	6.67	86.6 (1.3)
30	Dimethylsildenafil ^2^	1–500	0.9985	0.2	1.86	1.23	109.3 (2.5)
31	Diphenhydramine ^2^	1–500	0.9978	0.4	7.53	1.02	105.7 (4.9)
32	Methylprednisolone ^2^	5–500	0.9991	2	1.97	4.12	96.0 (1.5)
33	Carbamazepine ^1^	25–1000	0.9939	1	0.72	5.51	95.3 (4.9)
34	Dimenhydrinate ^2^	1–500	0.9964	0.5	3.40	1.75	83.8 (2.9)
35	Betamethasone ^2^	5–500	0.9994	2	0.62	0.82	84.2 (4.7)
36	Eplerenone ^1^	25–2000	0.9964	2	1.98	4.22	107.5 (4.9)
37	Acetaminotadalafil ^2^	5–500	0.9978	0.7	0.95	3.30	105.5 (1.7)
38	Dexamethasone ^2^	5–500	0.9998	1	2.01	8.05	89.7 (1.4)
39	Udenafil ^2^	5–500	0.9987	0.2	4.58	4.90	107.0 (5.6)
40	Promethazine ^2^	5–500	0.9963	2	3.55	2.74	104.1 (2.7)
41	Demethyltadalafil ^1^	25–500	0.9994	10	1.73	5.84	109.6 (1.3)
42	Piroxicam ^1^	25–500	0.9902	16	8.24	11.37	101.7 (1.9)
43	Paroxetine ^1^	25–500	0.9927	0.2	4.54	0.32	96.3 (2.7)
44	Beclomethasone ^2^	5–500	0.9992	0.9	4.32	2.74	95.2 (8.8)
45	4-Isopropylantipyrine ^1^	25–500	0.9974	0.2	0.93	10.15	104.7 (5.4)
46	Phenolphthalein ^1^	25–1000	0.9958	1	1.44	13.21	101.1 (0.8)
47	Ketorolac ^1^	25–1000	0.9957	2	0.17	0.90	101.1 (1.3)
48	Flunisolide ^2^	1–1000	0.9984	0.3	3.11	1.68	87.3 (4.9)
49	Sulindac ^1^	25–2000	0.9923	2	0.48	7.22	94.1 (2.8)
50	Cyproheptadine ^2^	1–500	0.9973	0.1	0.57	4.55	96.4 (1.5)
51	Levothyroxine ^1^	25–500	0.9940	2	1.08	10.54	105.6 (14.4)
52	Bisacodyl ^1^	25–500	0.9963	0.3	0.88	9.94	87.0 (9.4)
53	Boldenone ^2^	5–500	0.9997	0.1	3.88	3.02	110.5 (6.1)
54	Desulfovardenafil ^2^	5–500	0.9966	0.4	2.51	2.78	112.7 (1.5)
55	Benzylsildenafil ^2^	5–500	0.9988	2	5.03	5.57	88.0 (1.8)
56	Xanthoanthrafil ^2^	5–500	0.9969	3	3.40	3.84	116.3 (4.6)
57	Didesmethylsibutramine ^1^	25–500	0.9924	0.4	4.12	5.27	99.9 (0.1)
58	Prednisone-21-acetate ^2^	5–500	0.9987	1	3.96	4.84	91.5 (0.3)
59	Fexofenadine ^2^	5–500	0.9964	2	4.00	2.54	98.2 (1.2)
60	Fluoxetine HCl ^1^	25–2000	0.9908	0.2	0.75	0.44	93.8 (4.5)
61	Dapoxetine ^2^	5–500	0.9973	0.3	3.51	3.81	100.4 (5.2)
62	Mirodenafil ^2^	5–500	0.9973	0.7	3.12	7.42	108.6 (2.0)
63	Prednisolone-21-acetate ^1^	25–500	0.9997	7	4.37	5.53	94.7 (1.5)
64	Beclomethasone-21-Hemisuccinate ^1^	25–500	0.9995	8	1.99	3.64	100.1 (4.3)
65	Cortisone-21-acetate ^2^	5–500	0.9998	0.6	1.77	6.05	111.7 (0.3)
66	Sibutramine ^1^	25–500	0.9944	0.3	5.31	8.74	99.7 (1.6)
67	Sertraline HCl ^1^	25–500	0.9933	0.2	0.74	8.09	101.6 (9.3)
68	Homotadalafil ^1^	25–500	0.9997	8	2.44	6.88	112.5 (6.8)
69	Boldione ^2^	1–500	0.9988	0.1	3.24	1.73	81.6 (3.2)
70	Meloxicam ^1^	25–1000	0.9932	0.2	0.41	0.59	99.2 (0.6)
71	Mibolerone ^2^	5–500	0.9993	0.1	5.10	2.40	112.1 (6.5)
72	Danazol (M) ^2^	5–500	0.9997	0.2	1.98	1.35	82.0 (0.1)
73	Chlorosibutramine ^1^	25–500	0.9973	0.2	7.5	10.68	83.8 (10.9)
74	Spironolactone ^1^	25–2000	0.9925	4	4.22	1.98	94.1 (10.4)
75	Fluocinonide ^2^	5–500	0.9992	0.2	4.02	4.43	97.1 (7.2)
76	Calusterone ^2^	5–500	0.9980	0.1	1.98	6.00	99.4 (7.0)
77	Clostebol ^2^	5–500	0.9987	2	4.11	2.92	109.5 (8.3)
78	Cyclopentyltadalafil ^2^	5–500	0.9992	0.8	0.77	4.22	109.3 (3.8)
79	Chloropretadalafil ^2^	5–500	0.9997	0.1	1.30	3.39	93.6 (1.3)
80	Betamethasone-17-valerate ^2^	5–500	0.9994	0.8	2.13	5.51	99.3 (2.8)
81	Diclofenac ^1^	25–2000	0.9968	5	0.52	6.03	97.4 (2.3)
82	Indomethacin ^1^	25–2000	0.9932	10	0.94	14.65	104.7 (3.8)
83	Aceclofenac ^1^	25–2000	0.9919	5	1.02	2.14	87.2 (9.8)
84	Imidazosagatriazinone ^2^	5–500	0.9992	0.1	4.72	4.98	91.7 (1.5)
85	Terfenadine ^2^	5–500	0.9971	0.1	1.71	0.42	104.2 (2.1)
86	Phenylbutazone ^1^	50–2000	0.9917	6	1.08	10.08	102.3 (3.1)
87	Norbolethone ^2^	5–500	0.9999	0.1	1.84	5.20	90.8 (6.4)
88	Betamethasone-21-valerate ^2^	5–500	0.9995	3	2.31	4.43	114.6 (5.4)
89	Betamethasone dipropionate ^2^	5–500	0.9994	0.3	2.61	5.47	97.0 (1.1)
90	Beclomethasone dipropionate ^2^	5–500	0.9985	0.1	1.78	1.23	88.7 (2.5)
91	Rimonabant ^1^	25–2000	0.9963	0.2	4.96	4.09	94.1 (5.1)
92	Testosterone-17-propionate ^2^	5–500	0.9979	0.2	6.73	2.14	96.8 (2.8)

^1^ Precision and accuracy were evaluated at 200 ng/g level. ^2^ Precision and accuracy were evaluated at 50 ng/g level.

## Data Availability

Data available in a publicly accessible repository.

## Supplementary Materials

The following are available online at https://www.mdpi.com/article/10.3390/ph14060570/s1, Figure S1: Chemical structures of 25 erectile dysfunction drugs and their internal standards, Figure S2: Chemical structures of 12 antihistamines and their internal standard, Figure S3:Chemical structures of 22 steroids, anabolic steroids and their internal standards, Figure S4: Chemical structures of 12 NSAIDs, 4 diuretics and their internal standards, Figure S5: Chemical structures of 17 weight-loss drugs and their internal standards, Figure S6: Analytical procedures of three sample pretreatment methods for determination of illegal adulterants in soft-gel by UHPLC-Q/TOF-MS, Table S1: Penalty points for screening of 92 illegal adulterants in soft-gel-type dietary supplements using QuEChERS, EMR-Lipid, and DLLME, Table S2: Retention times, molecular formulae, and accurate mass measurements of 92 illegal adulterants obtained by UHPLC-Q/TOF-MS.
