# Supplementary material for: Efficient Matrix Cleanup of Soft-Gel-Type Dietary Supplements for Rapid Screening of 92 Illegal Adulterants Using EMR-Lipid dSPE and UHPLC-Q/TOF-MS
Source: Pharmaceuticals (Basel). 2021 Jun 15;14(6):570. doi: 10.3390/ph14060570 (PMC8232078; doi:10.3390/ph14060570)
Supplement: Supplementary file 1 [file pharmaceuticals-14-00570-s001.zip › 210525_Supplementary Table_EMR-Lipid.pdf]

Supplementary Table S1. Penalty points for screening of 92 illegal adulterants in soft-gel-type dietary supplements using QuEChERS, EMR-Lipid, and DLLME followed by UHPLC-Q/TOF-MS.

	Penalty points		
	**QuEChERS**	**EMR-Lipid**	**DLLME**
**Reagents**			
Acetonitrile	8	4	4
formic acid	1		
$MgSO_4$	0		
NaCl	0		
PSA	2		
GCB	1		
C18	0		
EMR-Lipid material kit		0	
De-ionized water		0	
Chloroform			3
**Instruments**			
Energy			
Ultrasonic	0	0	0
Vortex	0	0	0
Centrifuge	2	2	2
LC-Q/TOF-MS	3	3	3
Occupation hazard	0	0	0
Waste			
1 - 10 mL (g)	3	3	3
Recycling	0	0	0
Total penalty points	25	17	20
Analytical Eco-scale total score	75	83	80

According to Gałuszka et al. (2012).

PSA, primary secondary amine; GCB, graphitized carbon black

Supplementary Table S2. Retention times, molecular formulae, and accurate mass measurements of 92 illegal adulterants obtained by UHPLC-Q/TOF-MS.

No.	Analyte	RT (min)	Molecular formula	Theoretical m/z ion (Da)	Error (ppm)
1	Metformin	1.24	$C_4H_{11}N_5$	130.1087	3.23
2	Amiloride HCl	1.34	$C_6H_8ClN_7O$	230.0552	0.71
3	4-Dimethylaminoantipyrine	1.37	$C_{13}H_{17}N_3O$	232.1444	-0.27
4	Theophylline	1.38	$C_7H_8N_4O_2$	181.0720	0.55
5	Ephedrine HCl	1.43	$C_{10}H_{15}NO$	166.1227	-2.75
6	Acetaminophen	1.54	$C_8H_9NO_2$	152.0706	3.96
7	Triamterene	1.57	$C_{12}H_{11}N_7$	254.1149	-2.01
8	Captopril	2.00	$C_9H_{15}NO_3S$	218.0846	2.42
9	Yohimbin	2.27	$C_{21}H_{26}N_2O_3$	355.2016	2.04
10	Hydroxyvardenafil	2.35	$C_{23}H_{32}N_6O_5S$	505.2228	1.33
11	Thioquinapiperifil	2.39	$C_{24}H_{28}N_6OS$	449.2118	1.56
12	Bambuterol	2.54	$C_{18}H_{29}N_3O_5$	368.2180	0.58
13	Vardenafil	2.62	$C_{23}H_{32}N_6O_4S$	489.2279	0.12
14	Carbodenafil	2.71	$C_{24}H_{32}N_6O_3$	453.2609	1.70
15	Brompheniramine	2.99	$C_{16}H_{19}BrN_2$	319.0804	0.19
16	Bupropion HCl	3.08	$C_{13}H_{18}ClNO$	240.1150	-4.67
17	Triprolidine	3.16	$C_{19}H_{22}N_2$	279.1856	3.49
18	Norneovardenafil	3.25	$C_{18}H_{20}N_4O_4$	357.1577	2.32
19	Hongdenafil	3.43	$C_{25}H_{34}N_6O_3$	467.2765	0.88
20	Dimethylacetildenafil	3.54	$C_{25}H_{34}N_6O_3$	467.2765	1.35
21	Ketotifen fumarate salt	3.61	$C_{19}H_{19}NOS$	310.1260	1.62
22	Icariin	3.63	$C_{33}H_{40}O_{15}$	677.2440	-0.41
23	Astemizole	3.73	$C_{28}H_{31}FN_4O$	459.2555	0.78
24	Propranolol	3.85	$C_{16}H_{21}NO_2$	260.1645	0.45
25	Modafinil	3.86	$C_{15}H_{15}NO_2S$	296.0716	0.89
26	Oxohongdenafil	3.97	$C_{25}H_{32}N_6O_4$	481.2558	-0.22
27	Olopatadine	4.00	$C_{21}H_{23}NO_3$	338.1751	1.42
28	Sildenafil	4.31	$C_{22}H_{30}N_6O_4S$	475.2122	-1.09
29	Avanafil	4.42	$C_{23}H_{26}ClN_7O_3$	484.1858	1.15
30	Dimethylsildenafil	4.51	$C_{23}H_{32}N_6O_4S$	489.2279	-0.08
31	Diphenhydramine	4.57	$C_{17}H_{21}NO$	256.1696	2.00
32	Methylprednisolone	4.90	$C_{22}H_{30}O_5$	375.2166	1.35
33	Carbamazepine	4.90	$C_{15}H_{12}N_2O$	237.1023	-0.28
34	Dimenhydrinate	5.07	$C_{17}H_{21}NO$	256.1696	-2.05
35	Betamethasone	5.15	$C_{22}H_{29}FO_5$	393.2072	-0.30
36	Eplerenone	5.16	$C_{24}H_{30}O_6$	415.2115	1.23
37	Acetaminotadalafil	5.17	$C_{23}H_{20}N_4O_5$	433.1506	0.79
38	Dexamethasone	5.25	$C_{22}H_{29}FO_5$	393.2072	0.49
39	Udenafil	5.29	$C_{25}H_{36}N_6O_4S$	517.2592	-0.30
40	Promethazine	5.33	$C_{17}H_{20}N_2S$	285.1420	3.87
41	Demethyltadalafil	5.38	$C_{21}H_{17}N_3O_4$	376.1292	4.48
42	Piroxicam	5.39	$C_{15}H_{13}N_3O_4S$	332.0700	3.16
43	Paroxetine	5.48	$C_{19}H_{20}FNO_3$	330.1500	-2.15
44	Beclomethasone	5.50	$C_{22}H_{29}ClO_5$	409.1776	-1.42
45	4-Isopropylantipyrine	5.60	$C_{14}H_{18}N_2O$	231.1492	0.84
46	Phenolphthalein	5.63	$C_{20}H_{14}O_4$	319.0965	-0.69
47	Ketorolac	5.79	$C_{15}H_{13}NO_3$	256.0968	1.21
48	Flunisolide	5.96	$C_{24}H_{31}FO_6$	435.2177	1.50
49	Sulindac	5.97	$C_{20}H_{17}FO_3S$	357.0955	-0.80
50	Cyproheptadine	6.07	$C_{21}H_{21}N$	288.1747	1.33
51	Levothyroxine	6.07	$C_{15}H_{11}I_4NO_4$	777.694	1.01
52	Bisacodyl	6.1	$C_{22}H_{19}NO_4$	362.1387	-0.91
53	Boldenone	6.19	$C_{19}H_{26}O_2$	287.2006	6.13
54	Desulfovardenafil	6.33	$C_{17}H_{20}N_4O_2$	313.1659	2.27
55	Benzylsildenafil	6.44	$C_{28}H_{34}N_6O_4S$	551.2435	-0.38
56	Xanthoanthrafil	6.44	$C_{19}H_{23}N_3O_6$	390.166	0.95
57	Didesmethylsibutramine	6.58	$C_{15}H_{22}ClN$	252.1514	-2.19
58	Prednisone-21-acetate	6.68	$C_{23}H_{28}O_6$	401.1959	0.18
59	Fexofeadine	6.71	$C_{32}H_{39}NO_4$	502.2952	-2.24
60	Fluoxetine HCl	6.72	$C_{17}H_{18}F_3NO$	310.1414	1.06

Supplementary Table S2. (continued)

No.	Analyte	RT (min)	Molecular formula	Theoretical m/z ion (Da)	Error (ppm)
61	Dapoxetine	6.77	$C_{21}H_{23}NO$	306.1852	0.15
62	Mirodenafil	6.77	$C_{26}H_{37}N_5O_5S$	532.2588	3.60
63	Prednisolone-21-acetate	6.77	$C_{23}H_{30}O_6$	403.2115	2.50
64	Beclomethasone-21-hemisuccinate	6.78	$C_{26}H_{33}ClO_8$	509.1937	3.11
65	Cortisone-21-acetate	6.78	$C_{23}H_{30}O_6$	403.2115	0.30
66	Sibutramine	7.10	$C_{17}H_{26}ClN$	280.1827	-1.60
67	Sertraline HCl	7.10	$C_{17}H_{17}Cl_2N$	306.0811	-2.60
68	Homotadalafil	7.11	$C_{23}H_{21}N_3O_4$	404.1605	2.00
69	Boldione	7.15	$C_{19}H_{24}O_2$	285.1849	-4.17
70	Meloxicam	7.40	$C_{14}H_{13}N_3O_4S_2$	352.0421	0.69
71	Mibolerone	7.90	$C_{20}H_{30}O_2$	303.2319	0.55
72	Danazol (M)	8.00	$C_{21}H_{28}O_2$	313.2162	0.01
73	Chlorosibutramine	8.24	$C_{17}H_{25}Cl_2N$	314.1437	1.55
74	Spironolactone	8.30	$C_{24}H_{32}O_4S$	341.2111	0.30
75	Fluocinonide	8.83	$C_{26}H_{32}F_2O_7$	495.2189	-1.48
76	Calusterone	8.85	$C_{21}H_{32}O_2$	317.2475	0.03
77	Clostebol	8.98	$C_{19}H_{27}ClO_2$	323.1772	-1.16
78	Cyclopentyltadalafil	9.01	$C_{26}H_{25}N_3O_4$	444.1918	1.79
79	Chloropretadalafil	9.28	$C_{22}H_{19}ClN_2O_5$	427.1055	-3.71
80	Betamethasone-17-valerate	9.50	$C_{27}H_{37}FO_6$	477.2647	-1.08
81	Diclofenac	9.51	$C_{14}H_{11}Cl_2NO_2$	296.0240	1.60
82	Indomethacin	9.52	$C_{19}H_{16}ClNO_4$	358.0841	3.54
83	Aceclofenac	9.67	$C_{16}H_{13}Cl_2NO_4$	354.0295	-2.47
84	Imidazosagatriazinone	9.95	$C_{17}H_{20}N_4O_2$	313.1659	0.31
85	Terfenadine	10.15	$C_{32}H_41NO_2$	472.321	0.42
86	Phenylbutazone	10.16	$C_{19}H_{20}N_2O_2$	309.1598	2.46
87	Norbolethone	10.24	$C_{21}H_{32}O_2$	317.2475	0.38
88	Betamethasone-21-valerate	10.35	$C_{27}H_{37}FO_6$	477.2647	0.18
89	Betamethasone dipropionate	10.54	$C_{28}H_{37}FO_7$	505.2596	-0.60
90	Beclomethasone dipropionate	11.00	$C_{28}H_{37}ClO_7$	521.2301	-1.79
91	Rimonabant	12.24	$C_{21}H_{32}O_2$	463.0854	0.39
92	Testosterone-17-propionate	12.90	$C_{22}H_{32}O_3$	345.2424	-1.70
