# Supplementary material for: Efficient Matrix Cleanup of Soft-Gel-Type Dietary Supplements for Rapid Screening of 92 Illegal Adulterants Using EMR-Lipid dSPE and UHPLC-Q/TOF-MS
Source: Pharmaceuticals (Basel). 2021 Jun 15;14(6):570. doi: 10.3390/ph14060570 (PMC8232078; doi:10.3390/ph14060570)
Supplement: Supplementary file 1 [file pharmaceuticals-14-00570-s001.zip › 210525_Supplment_data_EMR-Lipid.pptx]

## Slide 1
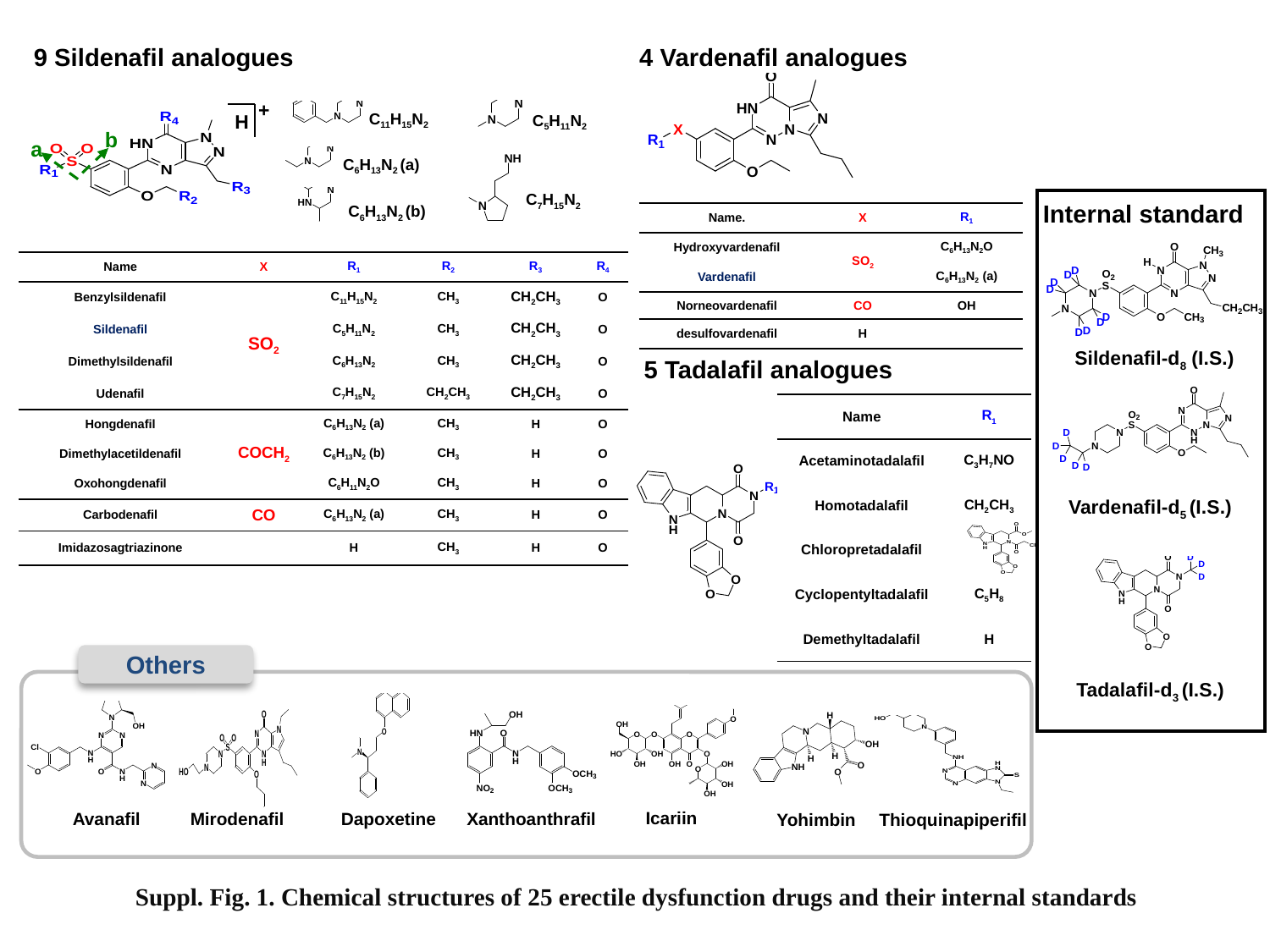

9 Sildenafil analogues
4 Vardenafil analogues
+
H
C11H15N2
C5H11N2
C6H13N2 (a)
C7H15N2
C6H13N2 (b)
b
a
Internal standard
Sildenafil-d8 (I.S.)
Vardenafil-d5 (I.S.)
Tadalafil-d3 (I.S.)
| Name. | X | R1 |
| --- | --- | --- |
| Hydroxyvardenafil | SO2 | C6H13N2O |
| Vardenafil | | C6H13N2 (a) |
| Norneovardenafil | CO | OH |
| desulfovardenafil | H | |
| Name | X | R1 | R2 | R3 | R4 |
| --- | --- | --- | --- | --- | --- |
| Benzylsildenafil | SO2 | C11H15N2 | CH3 | CH2CH3 | O |
| Sildenafil | | C5H11N2 | CH3 | CH2CH3 | O |
| Dimethylsildenafil | | C6H13N2 | CH3 | CH2CH3 | O |
| Udenafil | | C7H15N2 | CH2CH3 | CH2CH3 | O |
| Hongdenafil | COCH2 | C6H13N2 (a) | CH3 | H | O |
| Dimethylacetildenafil | | C6H13N2 (b) | CH3 | H | O |
| Oxohongdenafil | | C6H11N2O | CH3 | H | O |
| Carbodenafil | CO | C6H13N2 (a) | CH3 | H | O |
| Imidazosagtriazinone | | H | CH3 | H | O |
5 Tadalafil analogues
| Name | R1 |
| --- | --- |
| Acetaminotadalafil | C3H7NO |
| Homotadalafil | CH2CH3 |
| Chloropretadalafil | |
| Cyclopentyltadalafil | C5H8 |
| Demethyltadalafil | H |
Others
Dapoxetine
Avanafil
Icariin
Xanthoanthrafil
Yohimbin
Thioquinapiperifil
Mirodenafil
 Suppl. Fig. 1. Chemical structures of 25 erectile dysfunction drugs and their internal standards

## Slide 2
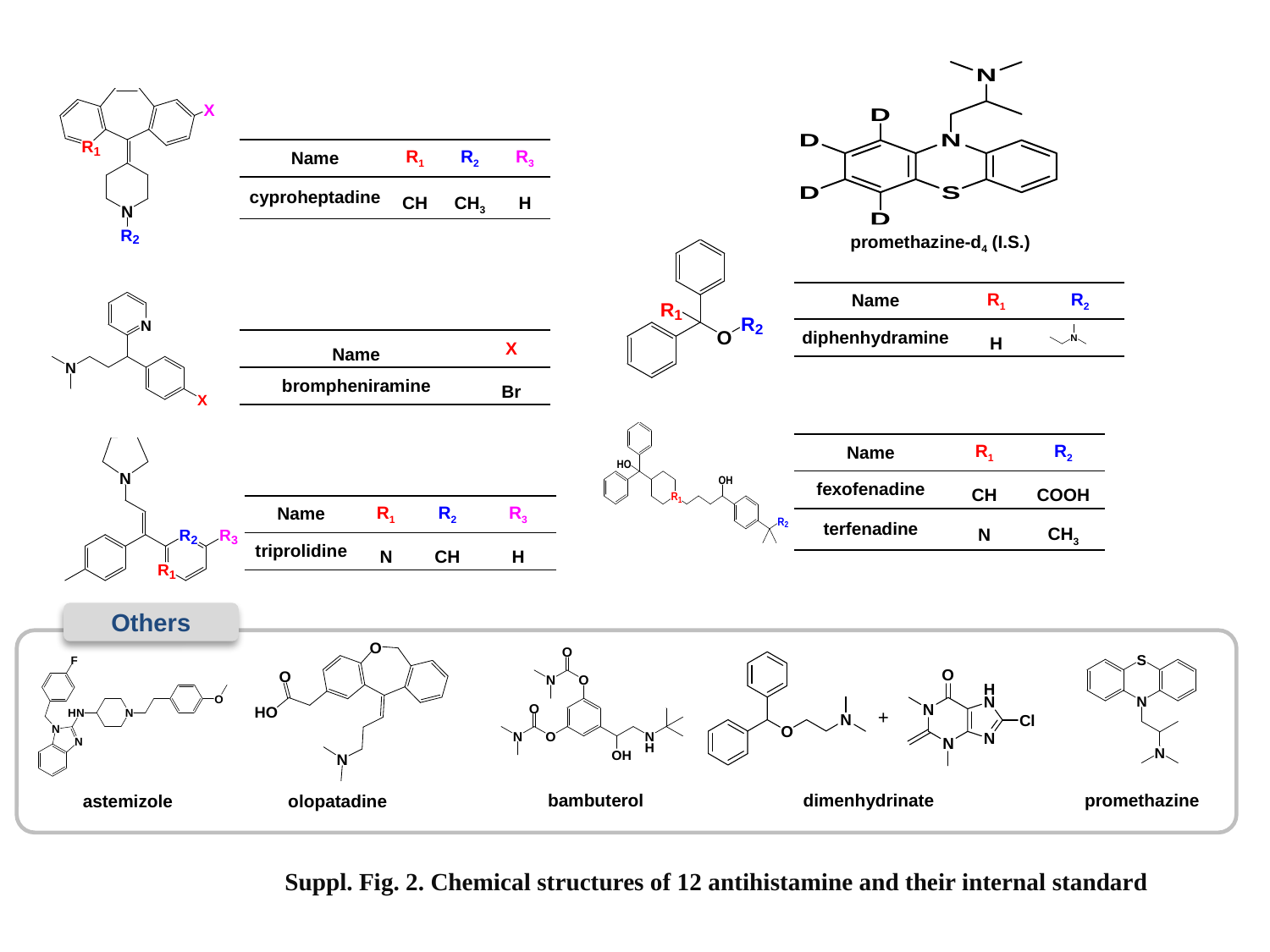

promethazine-d4 (I.S.)
| Name | R1 | R2 | R3 |
| --- | --- | --- | --- |
| cyproheptadine | CH | CH3 | H |
| Name | R1 | R2 |
| --- | --- | --- |
| diphenhydramine | H | |
| Name | X |
| --- | --- |
| brompheniramine | Br |
| Name | R1 | R2 |
| --- | --- | --- |
| fexofenadine | CH | COOH |
| terfenadine | N | CH3 |
| Name | R1 | R2 | R3 |
| --- | --- | --- | --- |
| triprolidine | N | CH | H |
Others
olopatadine
bambuterol
+
dimenhydrinate
astemizole
promethazine
 Suppl. Fig. 2. Chemical structures of 12 antihistamine and their internal standard

## Slide 3
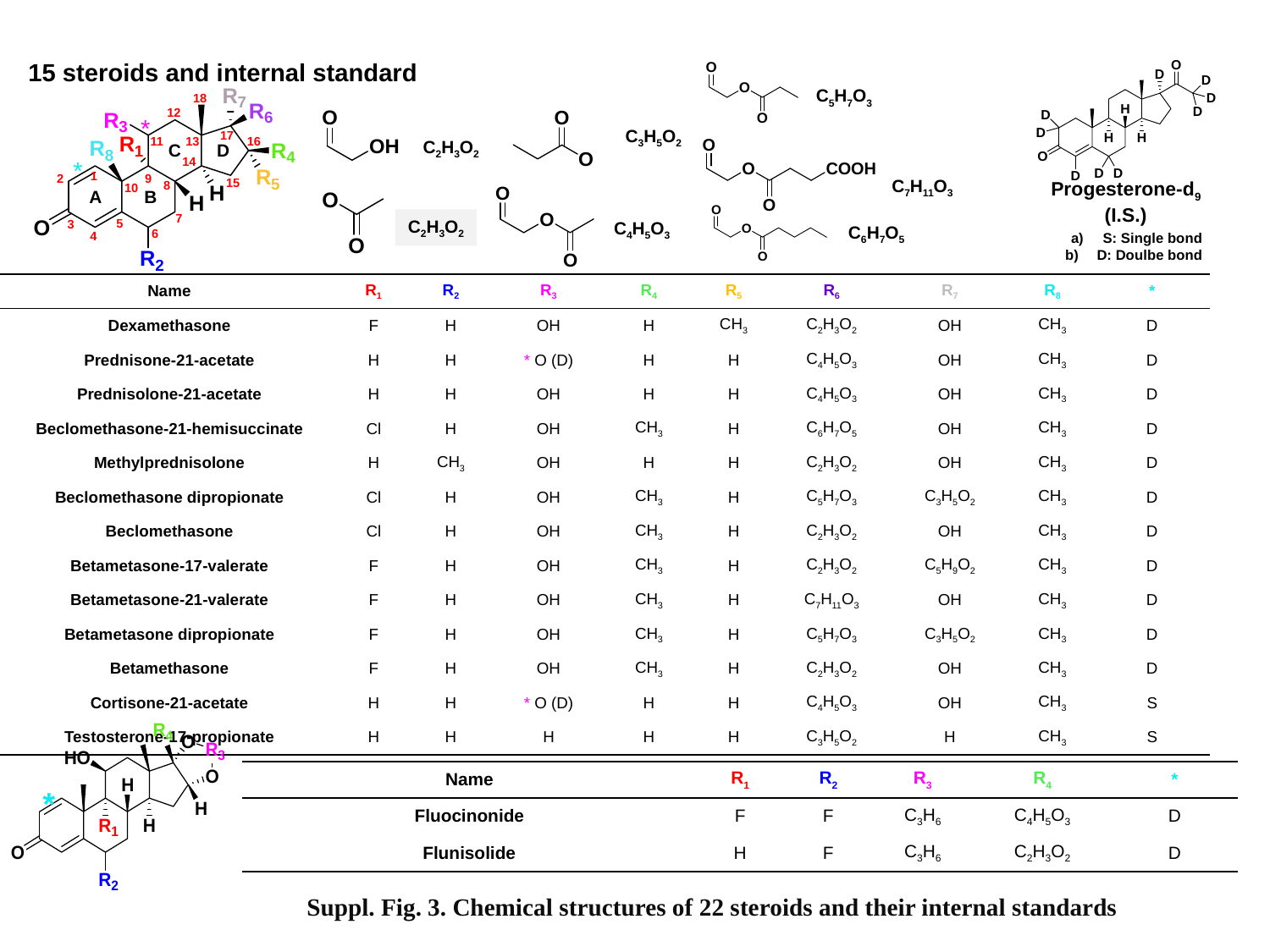

15 steroids and internal standard
C5H7O3
18
12
*
17
13
11
16
C
D
14
*
1
2
9
15
8
10
A
B
7
5
3
6
4
C3H5O2
C2H3O2
C7H11O3
Progesterone-d9 (I.S.)
C2H3O2
C4H5O3
C6H7O5
S: Single bond
D: Doulbe bond
| Name | R1 | R2 | R3 | R4 | R5 | R6 | R7 | R8 | \* |
| --- | --- | --- | --- | --- | --- | --- | --- | --- | --- |
| Dexamethasone | F | H | OH | H | CH3 | C2H3O2 | OH | CH3 | D |
| Prednisone-21-acetate | H | H | \* O (D) | H | H | C4H5O3 | OH | CH3 | D |
| Prednisolone-21-acetate | H | H | OH | H | H | C4H5O3 | OH | CH3 | D |
| Beclomethasone-21-hemisuccinate | Cl | H | OH | CH3 | H | C6H7O5 | OH | CH3 | D |
| Methylprednisolone | H | CH3 | OH | H | H | C2H3O2 | OH | CH3 | D |
| Beclomethasone dipropionate | Cl | H | OH | CH3 | H | C5H7O3 | C3H5O2 | CH3 | D |
| Beclomethasone | Cl | H | OH | CH3 | H | C2H3O2 | OH | CH3 | D |
| Betametasone-17-valerate | F | H | OH | CH3 | H | C2H3O2 | C5H9O2 | CH3 | D |
| Betametasone-21-valerate | F | H | OH | CH3 | H | C7H11O3 | OH | CH3 | D |
| Betametasone dipropionate | F | H | OH | CH3 | H | C5H7O3 | C3H5O2 | CH3 | D |
| Betamethasone | F | H | OH | CH3 | H | C2H3O2 | OH | CH3 | D |
| Cortisone-21-acetate | H | H | \* O (D) | H | H | C4H5O3 | OH | CH3 | S |
| Testosterone-17-propionate | H | H | H | H | H | C3H5O2 | H | CH3 | S |
*
| Name | R1 | R2 | R3 | R4 | \* |
| --- | --- | --- | --- | --- | --- |
| Fluocinonide | F | F | C3H6 | C4H5O3 | D |
| Flunisolide | H | F | C3H6 | C2H3O2 | D |
 Suppl. Fig. 3. Chemical structures of 22 steroids and their internal standards

## Slide 4
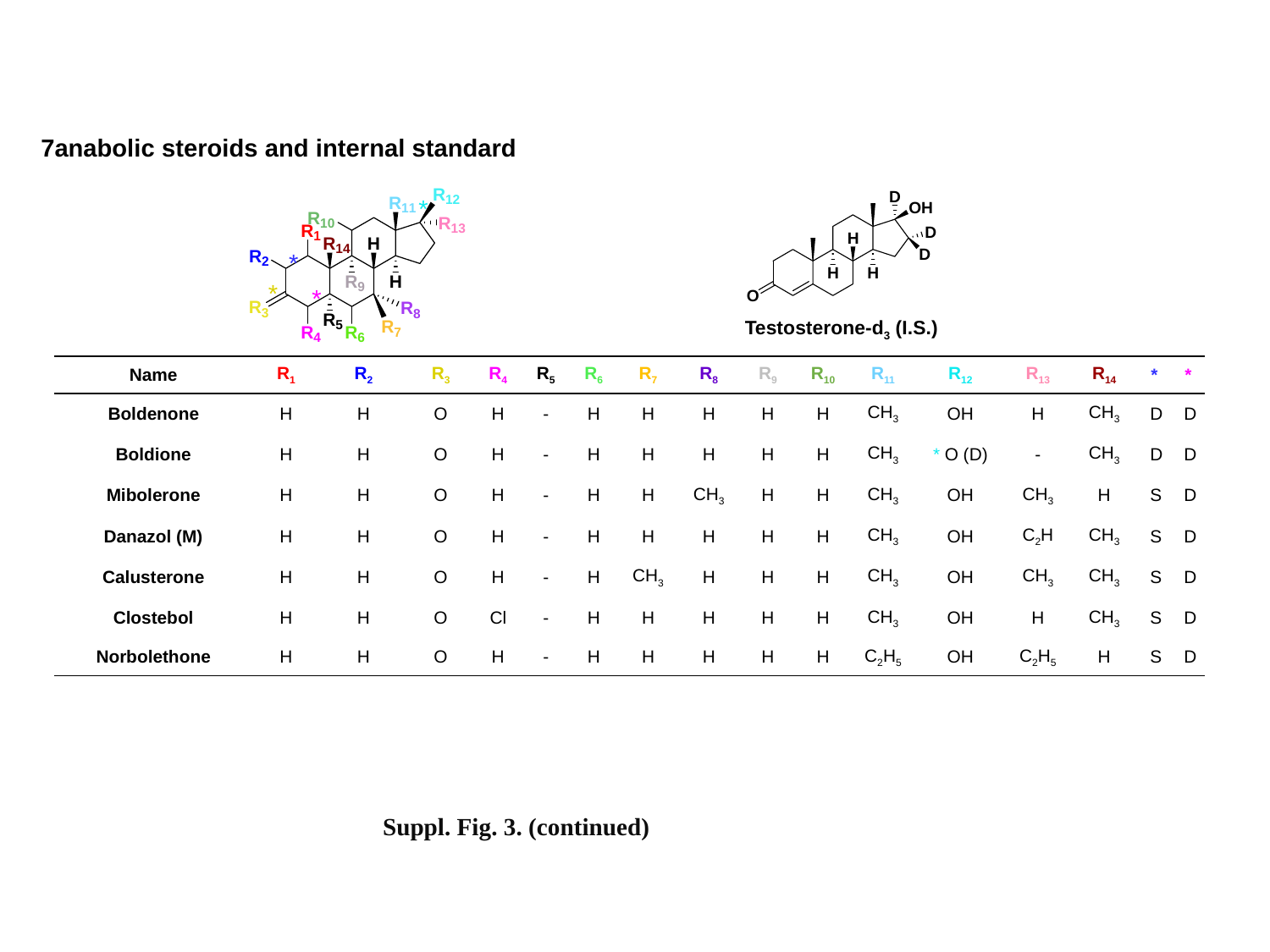

7anabolic steroids and internal standard
*
*
*
*
Testosterone-d3 (I.S.)
| Name | R1 | R2 | R3 | R4 | R5 | R6 | R7 | R8 | R9 | R10 | R11 | R12 | R13 | R14 | \* | \* |
| --- | --- | --- | --- | --- | --- | --- | --- | --- | --- | --- | --- | --- | --- | --- | --- | --- |
| Boldenone | H | H | O | H | - | H | H | H | H | H | CH3 | OH | H | CH3 | D | D |
| Boldione | H | H | O | H | - | H | H | H | H | H | CH3 | \* O (D) | - | CH3 | D | D |
| Mibolerone | H | H | O | H | - | H | H | CH3 | H | H | CH3 | OH | CH3 | H | S | D |
| Danazol (M) | H | H | O | H | - | H | H | H | H | H | CH3 | OH | C2H | CH3 | S | D |
| Calusterone | H | H | O | H | - | H | CH3 | H | H | H | CH3 | OH | CH3 | CH3 | S | D |
| Clostebol | H | H | O | Cl | - | H | H | H | H | H | CH3 | OH | H | CH3 | S | D |
| Norbolethone | H | H | O | H | - | H | H | H | H | H | C2H5 | OH | C2H5 | H | S | D |
 Suppl. Fig. 3. (continued)

## Slide 5
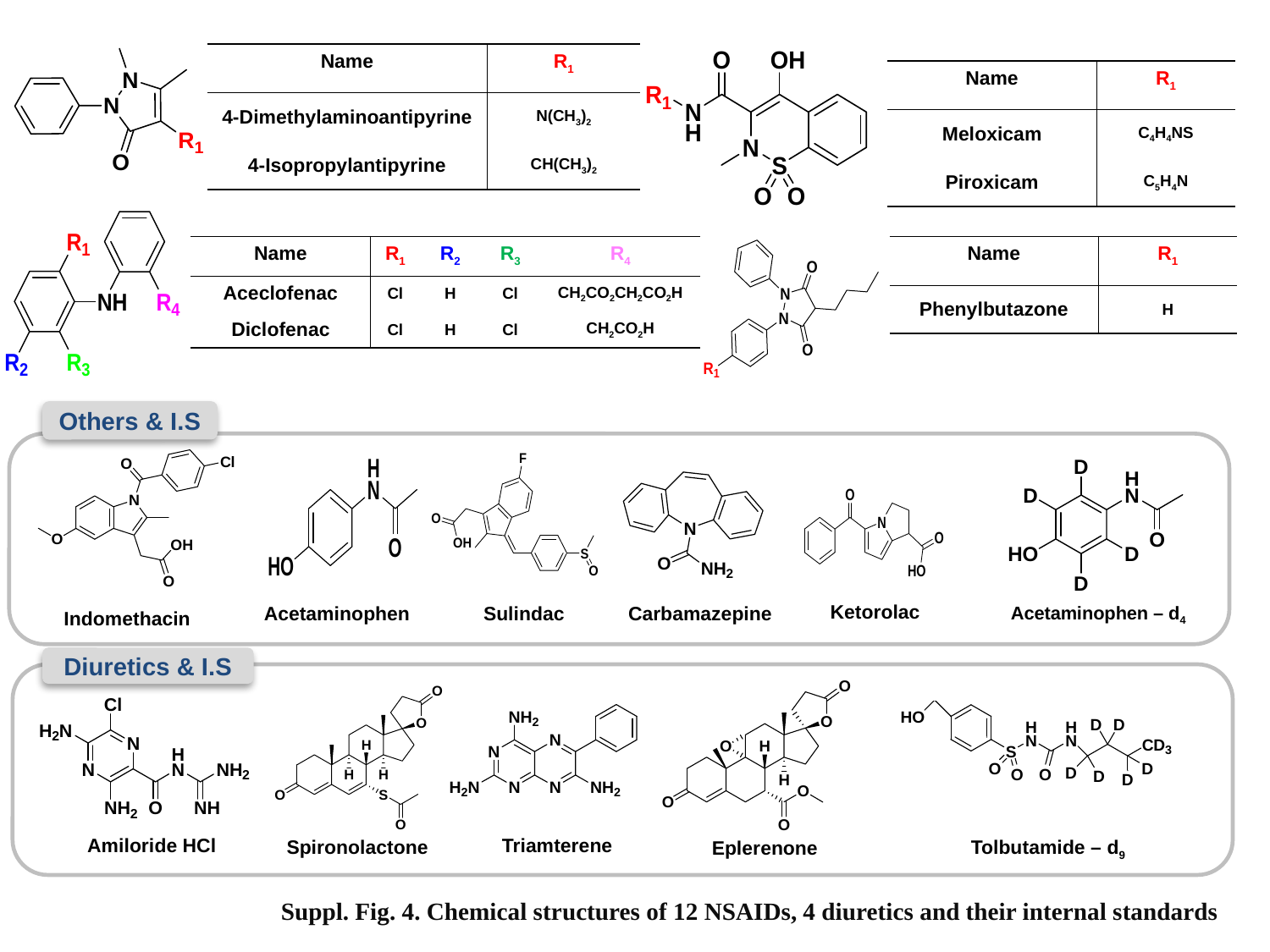

| Name | R1 |
| --- | --- |
| 4-Dimethylaminoantipyrine | N(CH3)2 |
| 4-Isopropylantipyrine | CH(CH3)2 |
| Name | R1 |
| --- | --- |
| Meloxicam | C4H4NS |
| Piroxicam | C5H4N |
| Name | R1 | R2 | R3 | R4 |
| --- | --- | --- | --- | --- |
| Aceclofenac | Cl | H | Cl | CH2CO2CH2CO2H |
| Diclofenac | Cl | H | Cl | CH2CO2H |
| Name | R1 |
| --- | --- |
| Phenylbutazone | H |
Others & I.S
Ketorolac
Acetaminophen – d4
Acetaminophen
Sulindac
Carbamazepine
Indomethacin
Diuretics & I.S
Triamterene
Amiloride HCl
Spironolactone
Tolbutamide – d9
Eplerenone
 Suppl. Fig. 4. Chemical structures of 12 NSAIDs, 4 diuretics and their internal standards

## Slide 6
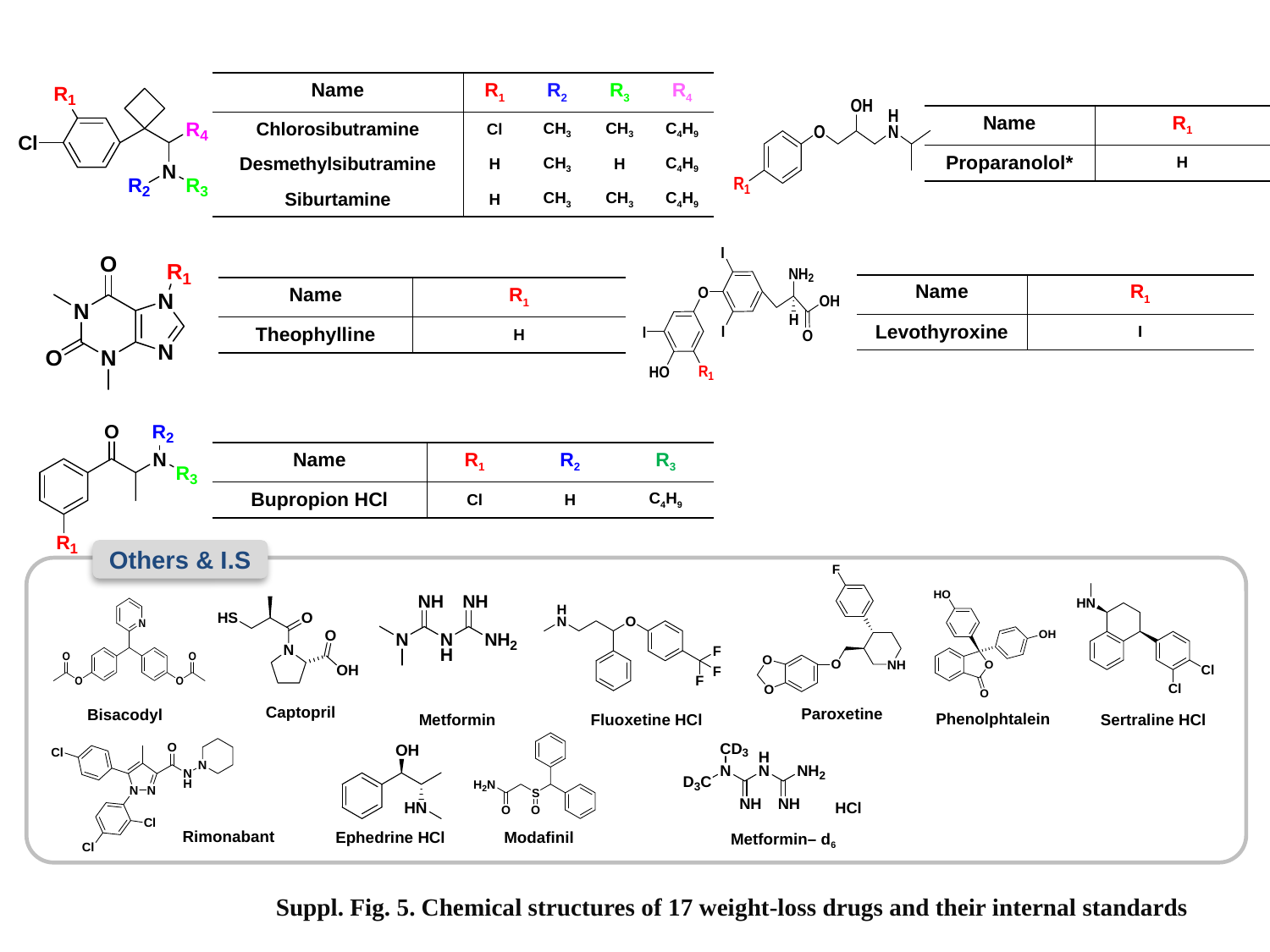

| Name | R1 | R2 | R3 | R4 |
| --- | --- | --- | --- | --- |
| Chlorosibutramine | Cl | CH3 | CH3 | C4H9 |
| Desmethylsibutramine | H | CH3 | H | C4H9 |
| Siburtamine | H | CH3 | CH3 | C4H9 |
| Name | R1 |
| --- | --- |
| Proparanolol\* | H |
| Name | R1 |
| --- | --- |
| Levothyroxine | I |
| Name | R1 |
| --- | --- |
| Theophylline | H |
| Name | R1 | R2 | R3 |
| --- | --- | --- | --- |
| Bupropion HCl | Cl | H | C4H9 |
Others & I.S
Captopril
Paroxetine
Bisacodyl
Phenolphtalein
Fluoxetine HCl
Sertraline HCl
Metformin
Rimonabant
Modafinil
Ephedrine HCl
Metformin– d6
 Suppl. Fig. 5. Chemical structures of 17 weight-loss drugs and their internal standards

## Slide 7
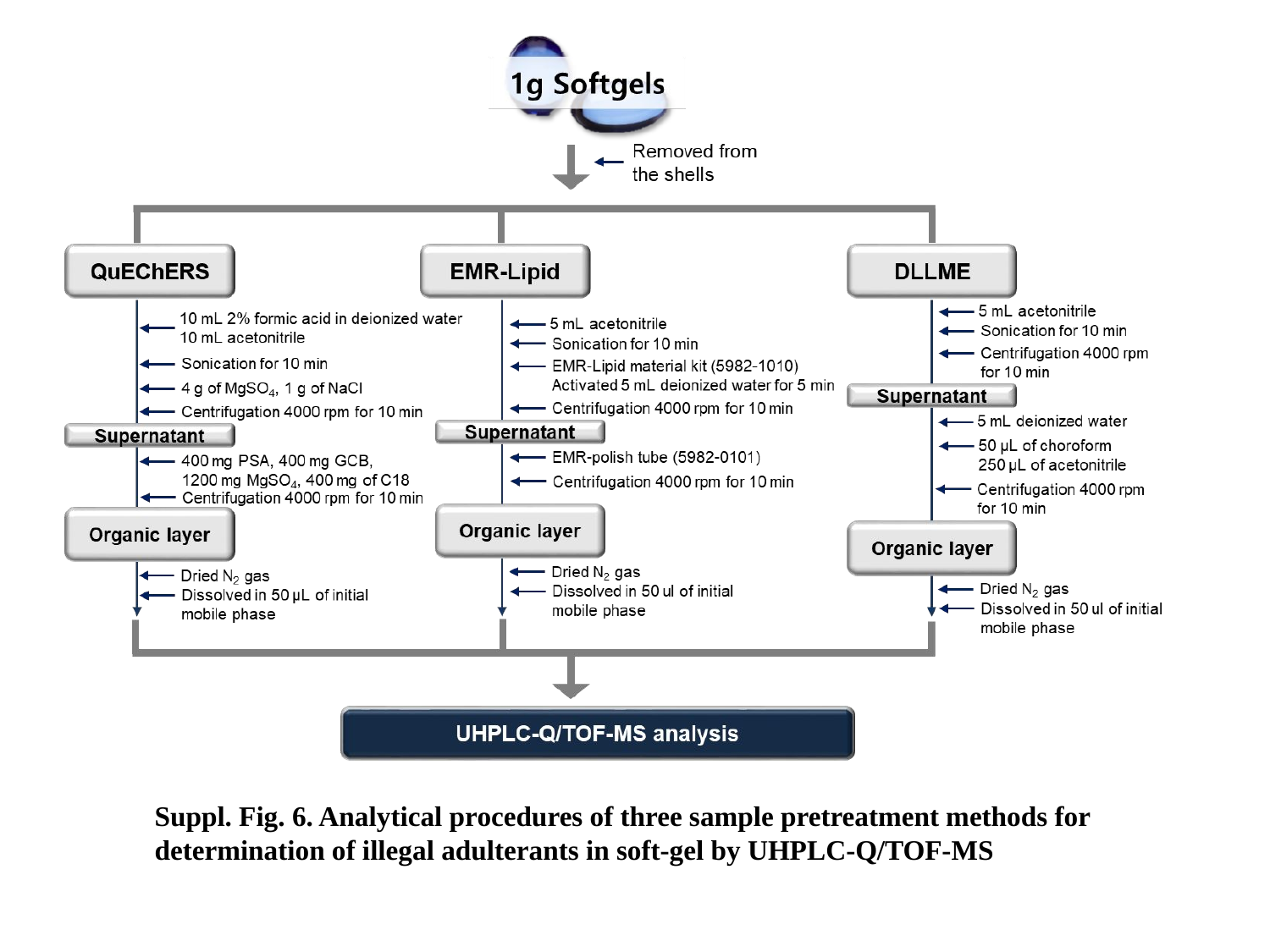

Suppl. Fig. 6. Analytical procedures of three sample pretreatment methods for determination of illegal adulterants in soft-gel by UHPLC-Q/TOF-MS
